# Supplementary material for: Ancestral neuronal receptors are bacterial accessory toxins
Source: Nat Commun. 2026 Feb 14;17:2753. doi: 10.1038/s41467-026-69246-x (PMC13018210; doi:10.1038/s41467-026-69246-x)
Supplement: Supplementary file 1 — Supplementary Information [file 41467_2026_69246_MOESM1_ESM.pdf]

Supplementary information for Ancestral neuronal receptors are bacterial accessory toxins

Finaritra Raoelijaona[1], Joanna Szczepaniak[1]†, Adrien Schahl[2]†, James E Bray[3]†, Jin Zhou[1]†, Lindsay Baker[1], Kamel El Omari[4], Edward Lowe[1], Yu Shang Low[6], Chandra M. Rodriguez[7], Michael J. Landsberg[6], J. Shaun Lott[7,8] Colin Kleanthous[1], Matthieu Chavent[2,5]*, Martin CJ Maiden[3]*, Elena Seiradake[1]*

Corresponding authors: elena.seiradake@bioch.ox.ac.uk, martin.maiden@biology.ox.ac.uk, matthieu.chavent@univ-tlse3.fr

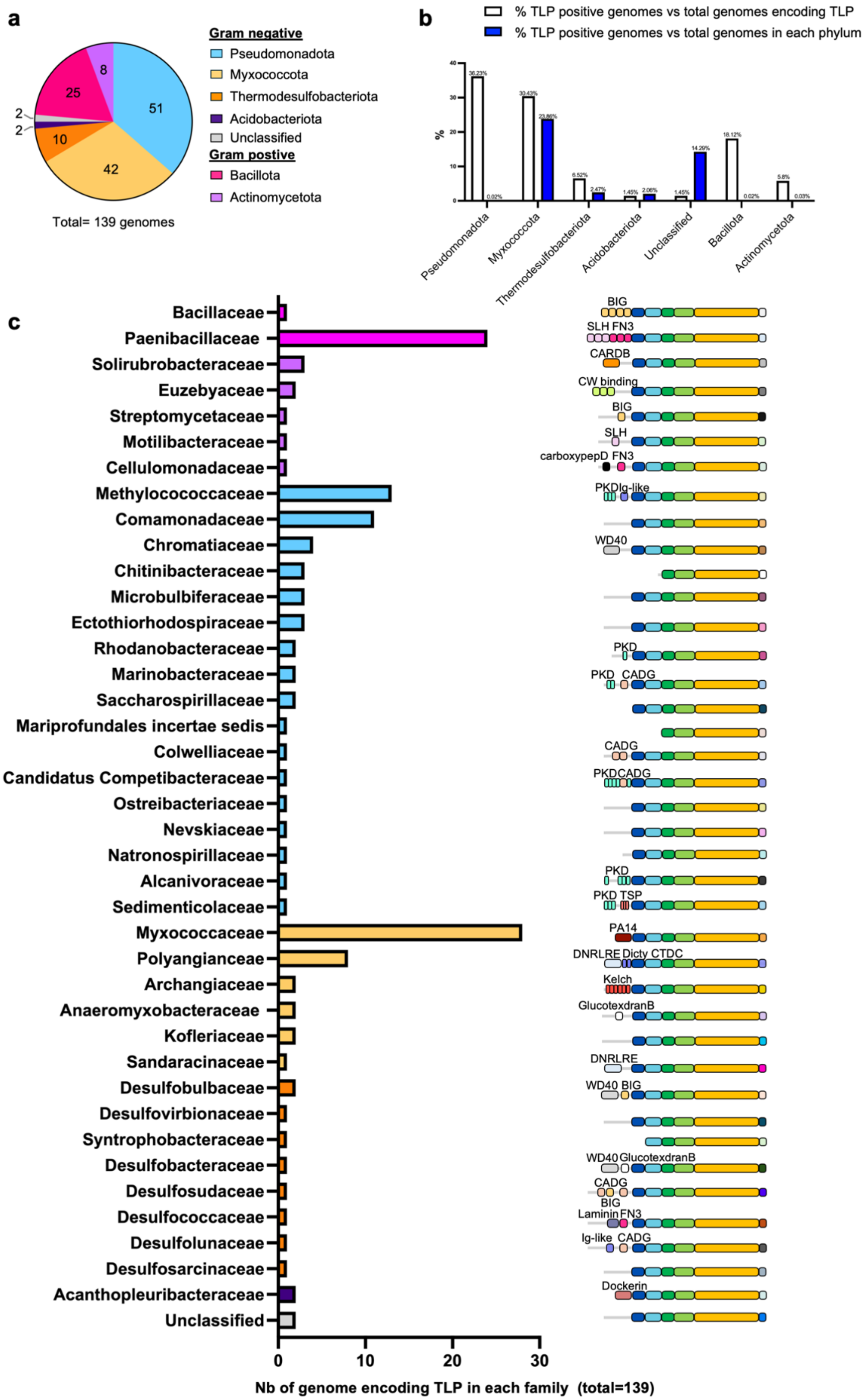
a
Gram negative
Pseudomonadota
Myxococcota
Thermodesulfobacteriota
Acidobacteriota
Unclassified
Gram postive
Bacillota
Actinomycetota
51
42
10
2
2
25
8
Total= 139 genomes
b
% TLP positive genomes vs total genomes encoding TLP
% TLP positive genomes vs total genomes in each phylum
%
Pseudomonadota
Myxococcota
Thermodesulfobacteriota
Acidobacteriota
Unclassified
Bacillota
Actinomycetota
c
Bacillaceae
Paenibacillaceae
Solirubrobacteraceae
Euzebyaceae
Streptomycetaceae
Motilibacteraceae
Cellulomonadaceae
Methylococcaceae
Comamonadaceae
Chromatiaceae
Chitinibacteraceae
Microbulbiferaceae
Ectothiorhodospiraceae
Rhodanobacteraceae
Marinobacteraceae
Saccharospirillaceae
Mariprofundales incertae sedis
Colwelliaceae
Candidatus Competibacteraceae
Ostreibacteriaceae
Nevskiaceae
Natronospirillaceae
Alcanivoraceae
Sedimenticolaceae
Myxococcaceae
Polyangiaceae
Archangiaceae
Anaeromyxobacteraceae
Kofleriaceae
Sandaracinaceae
Desulfobulbaceae
Desulfovirbionaceae
Syntrophobacteraceae
Desulfobacteraceae
Desulfosudaceae
Desulfococcaceae
Desulfolunaceae
Desulfosarcinaceae
Acanthopleuribacteraceae
Unclassified
0
10
20
30
Nb of genome encoding TLP in each family (total=139)
BIG
SLH FN3
CARDB
CW binding
BIG
SLH
carboxypepD FN3
PKDIg-like
WD40
PKD
PKD CADG
CADG
PKDCADG
PKD
PKD TSP
PA14
DNRLRE Dicty CTDC
Kelch
GlucotexdranB
DNRLRE
WD40 BIG
WD40 GlucotexdranB
CADG
BIG
Laminin FN3
Ig-like CADG
Dockerin

Supplementary Fig. 1 | TLP genes are enriched in phyla exhibiting complex social lifestyles.

a Pie chart showing the distribution of bacterial TLP positive genomes across different phyla found in the PubMLST multispecies database [1]. Unclassified refers to an *Olavius algarvensis* associated proteobacterium Delta 3. **b** Histogram displaying how many TLP-encoding genomes were found in each phylum, as a percentage of all TLP-encoding genomes (white bars), and as a percentage of all genomes in the respective phylum (blue bars). **c** Left: analysis of TLP-encoding genomes for each family, showing the number of TLP-encoding genomes found in each family. Bars are colored according to the corresponding phylum: Bacillota (magenta), Actinomycetota (purple), Pseudomonadota (blue), Myxococcota (yellow), Thermodesulfobacteriota (orange), Acidobacteriota (dark purple), Unclassified (grey). Right: TLP domain organisation in each family. FN-plug (forest green), NHL (lime green), YD-shell (triple gradient color: yellow-orange-red: from N-terminal to C-terminal of the YD-shell), TIP (light blue), TTR (dark blue). The CTD is coloured in multiple colours to reflect hypervariability within this region. The variable N-terminal region was annotated based on domain conservation prediction using Interproscan and CD search servers. BIG (Bacterial immunoglobulin-like), SLH (S-layer), FN3 (Fibronectin type-3), CARDB (Cell adhesion related domain found in bacteria), CW binding (Cell wall binding domain), CarboxypepD (carboxypeptidase-like regulatory domain), PKD (Polycystic kidney disease domain), Ig-like (Immunoglobulin-like domain), WD40 (WD-repeat proteins), CADG (Dystroglycan-type cadherin-like), Dicty_CTDC (Dictyostelium (slime mold) repeat).

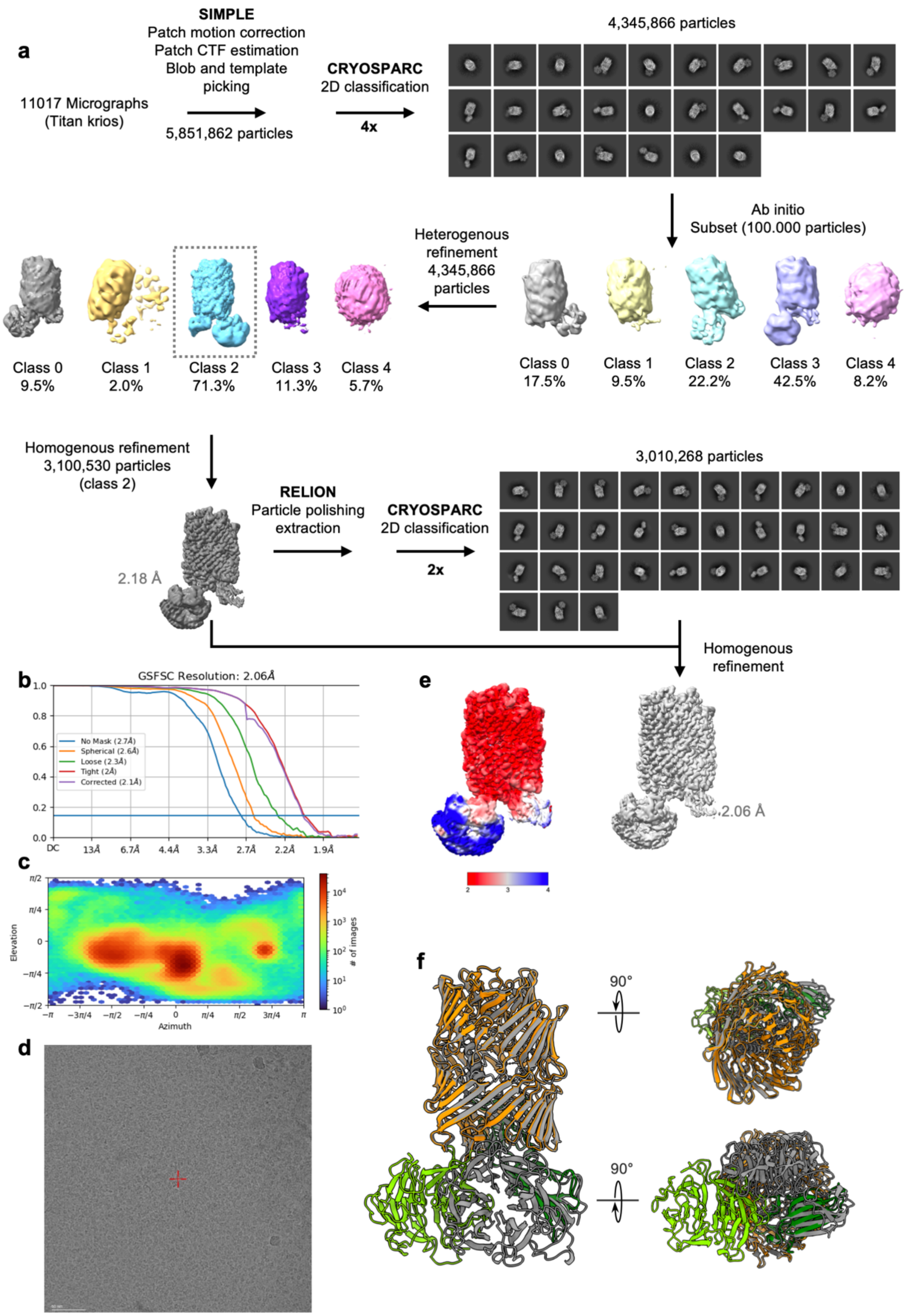

a
SIMPLE
Patch motion correction
Patch CTF estimation
Blob and template picking
11017 Micrographs (Titan krios)
5,851,862 particles
CRYOSPARC
2D classification
4x
4,345,866 particles
Ab initio
Subset (100.000 particles)
Heterogenous refinement
4,345,866 particles
Class 0 17.5%
Class 1 9.5%
Class 2 22.2%
Class 3 42.5%
Class 4 8.2%
Class 0 9.5%
Class 1 2.0%
Class 2 71.3%
Class 3 11.3%
Class 4 5.7%
Homogenous refinement
3,100,530 particles
(class 2)
2.18 Å
RELION
Particle polishing
extraction
CRYOSPARC
2D classification
2x
3,010,268 particles
Homogenous refinement
b
GSFSC Resolution: 2.06Å
No Mask (2.7Å)
Spherical (2.6Å)
Loose (2.3Å)
Tight (2Å)
Corrected (2.1Å)
c
Elevation
Azimuth
# of images
d
e
2.06 Å
f
90°
90°

Supplementary Fig. 2 | *Bacillus inaquosorum* TLP single particle Cryo-EM data processing.

a Summary of the image processing workflow. **b** Gold-standard Fourier Shell Correlation (FSC) curves for global resolution estimation. **c** Orientation distribution plot. **d** Representative micrograph. **e** Local resolution estimate of the volume. **f** Overlay of the predicted AlphaFold2 [2] model *Bi*TLP (grey) and the model following refinement into the cryo-EM map colored according to its domain composition: FN-plug (lime green), NHL (forest green), YD-shell (orange), CTD (light grey)

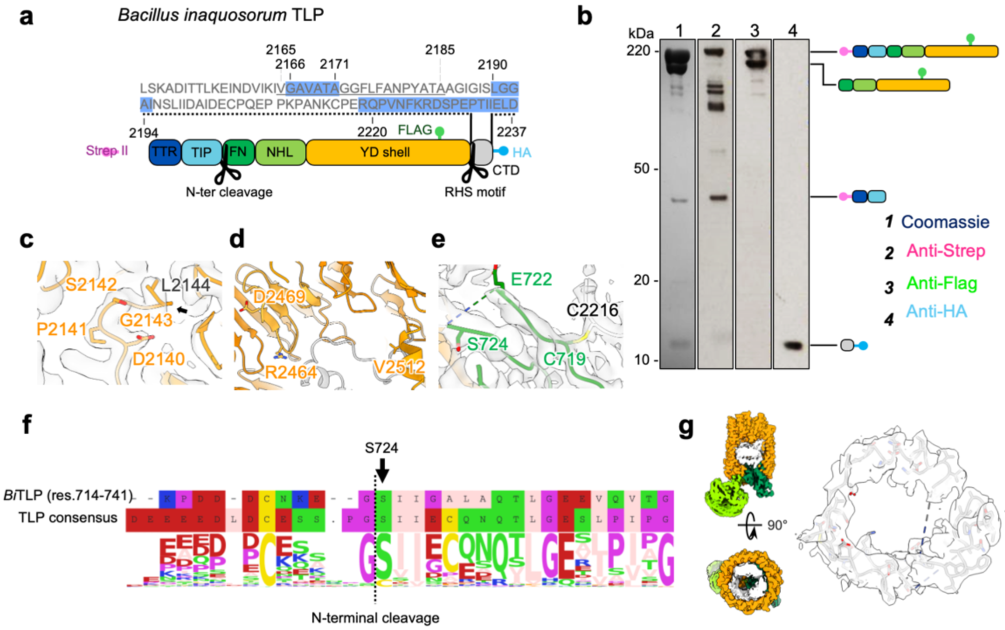

a
Bacillus inaquosorum TLP
2165 2166 2171 2185 2190
LSKADITTLKEINDVIKIVGAVATAGGFLFANPYATAAGIGISLGG
AINSLIIDAIDECPQEP PKPANKCPERQPVNFKRDSPEPTIIELD
2194 2220 2237
FLAG
Strep II
TTR TIP FN NHL YD shell
HA
CTD
N-ter cleavage
RHS motif
b
kDa 1 2 3 4
220
50
20
10
1 Coomassie
2 Anti-Strep
3 Anti-Flag
4 Anti-HA
c
S2142 L2144 P2141 G2143 D2140
d
D2469 R2464 V2512
e
E722 C2216 S724 C719
f
S724
BiTLP (res.714-741)
TLP consensus
N-terminal cleavage
g
90°

Supplementary Fig. 3| *Bacillus inaquosorum* TLP shares characteristics with other RHS/YD proteins.

a To visualize different *Bi*TLP fragments, we produced a construct with an N-terminal strepII tag, a central FLAG-tag (within the RHS associated core) and a C-terminal HA-tag. The sequence corresponding to the cleaved C-terminal domain (CTD) is shown in grey, with the residues not resolved in our structural model highlighted in blue. The predicted transmembrane region is underlined in grey. **b** Following purification and size exclusion chromatography, tagged *Bi*TLP protein (see panel **a**) was analysed by SDS-PAGE. Proteins were either stained with Coomassie blue (lane 1) or transferred to a membrane for western blotting (lanes 2-4). In lane 1, four fragments are seen: a double band at ~220 kDa, one band at ~45 kDa, and a band at ~10 kDa. Immunoblotting of the same sample with anti-Strep (lane 2), anti-Flag (lane 3), and anti-HA (lane 4) led to identification of different *Bi*TLP fragments based on the predicted molecular weight and tag. **c** Zoomed view of the *Bi*TLP map (silver) at the conserved RHS cleavage motif. The cleavage occurs after L2144, where the break in the map is indicated by a black arrow. **d** Focused view of chicken Ten-2 'inactive' auto aspartyl protease catalytic site, where the second aspartate residue is replaced by valine (V2512). Ten-2 YD-shell (orange) forms a continuous protein chain with the CTD (light grey). **e** Zoomed view of region between residues E722 and S724 showing absence of map density. **f** Comparison between *Bi*TLP and a consensus sequence derived from all members of the TLP family, generated using MSAclass logo web server [3]. **g** Left: Section views of the *Bi*TLP map, colored according to the structured domains: FN-plug (forest green), NHL (lime green), YD-shell (orange), CTD (light grey). Right: Partially built fragments of the *Bi*TLP CTD within the map (silver). The CTD residues are shown in stick.

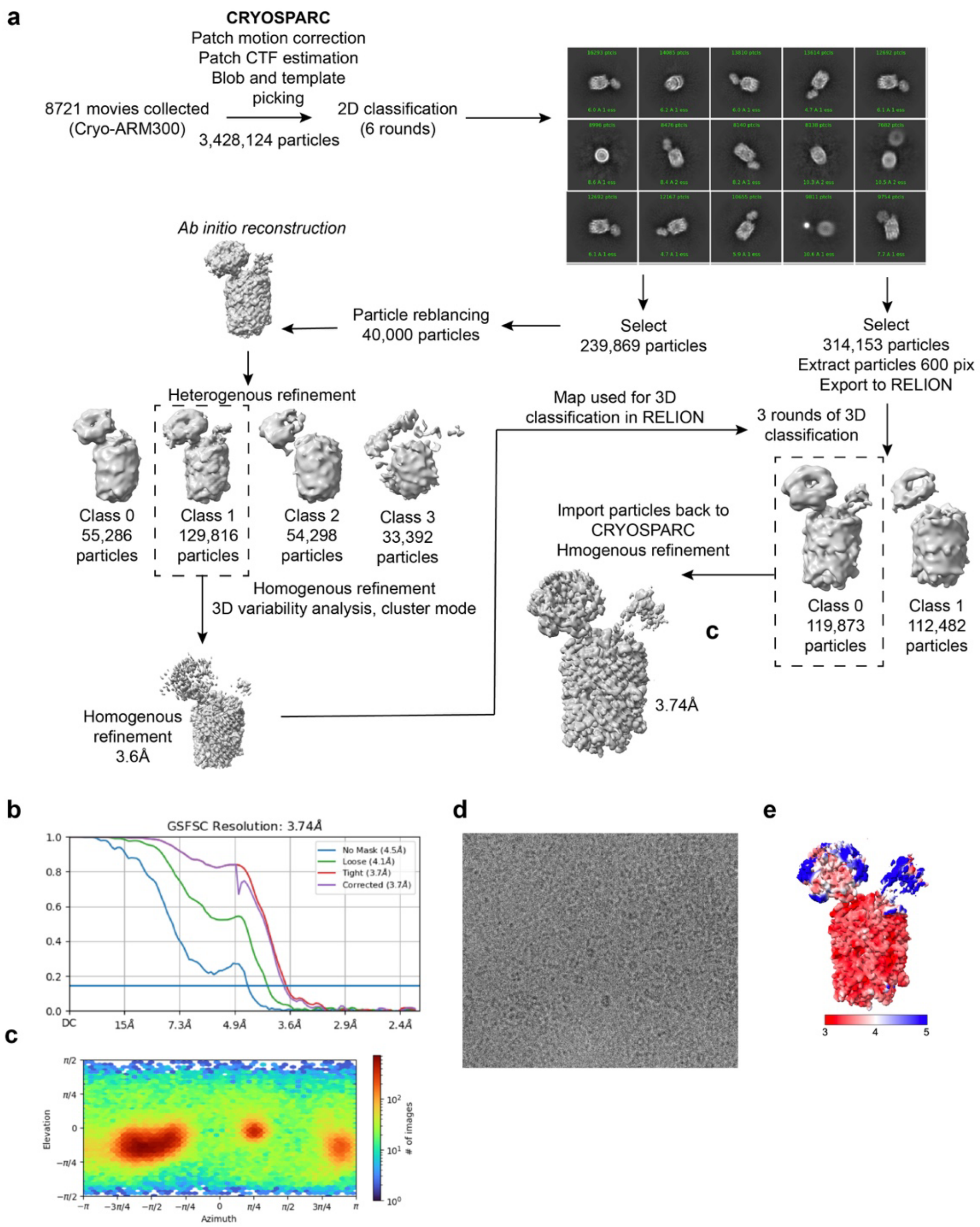
a
CRYOSPARC
Patch motion correction
Patch CTF estimation
Blob and template picking
8721 movies collected (Cryo-ARM300)
3,428,124 particles
2D classification (6 rounds)
Ab initio reconstruction
Particle reblancing
40,000 particles
Select
239,869 particles
Select
314,153 particles
Extract particles 600 pix
Export to RELION
Heterogenous refinement
Class 0
55,286 particles
Class 1
129,816 particles
Class 2
54,298 particles
Class 3
33,392 particles
Map used for 3D classification in RELION
3 rounds of 3D classification
Import particles back to CRYOSPARC
Hmogenous refinement
Homogenous refinement
3D variability analysis, cluster mode
Class 0
119,873 particles
Class 1
112,482 particles
c
3.74Å
Homogenous refinement
3.6Å
b
GSFSC Resolution: 3.74Å
No Mask (4.5Å)
Loose (4.1Å)
Tight (3.7Å)
Corrected (3.7Å)
c
Elevation
Azimuth
# of images
d
e

Supplementary Fig. 4 | Full length *Bacillus inaquosorum* TLP single particle Cryo-EM data processing.

a Summary of the image processing workflow. **b** Gold-standard Fourier Shell Correlation (FSC) curves for global resolution estimation. **c** Orientation distribution plot. **d** Representative micrograph. **e** Local resolution estimate of the volume.

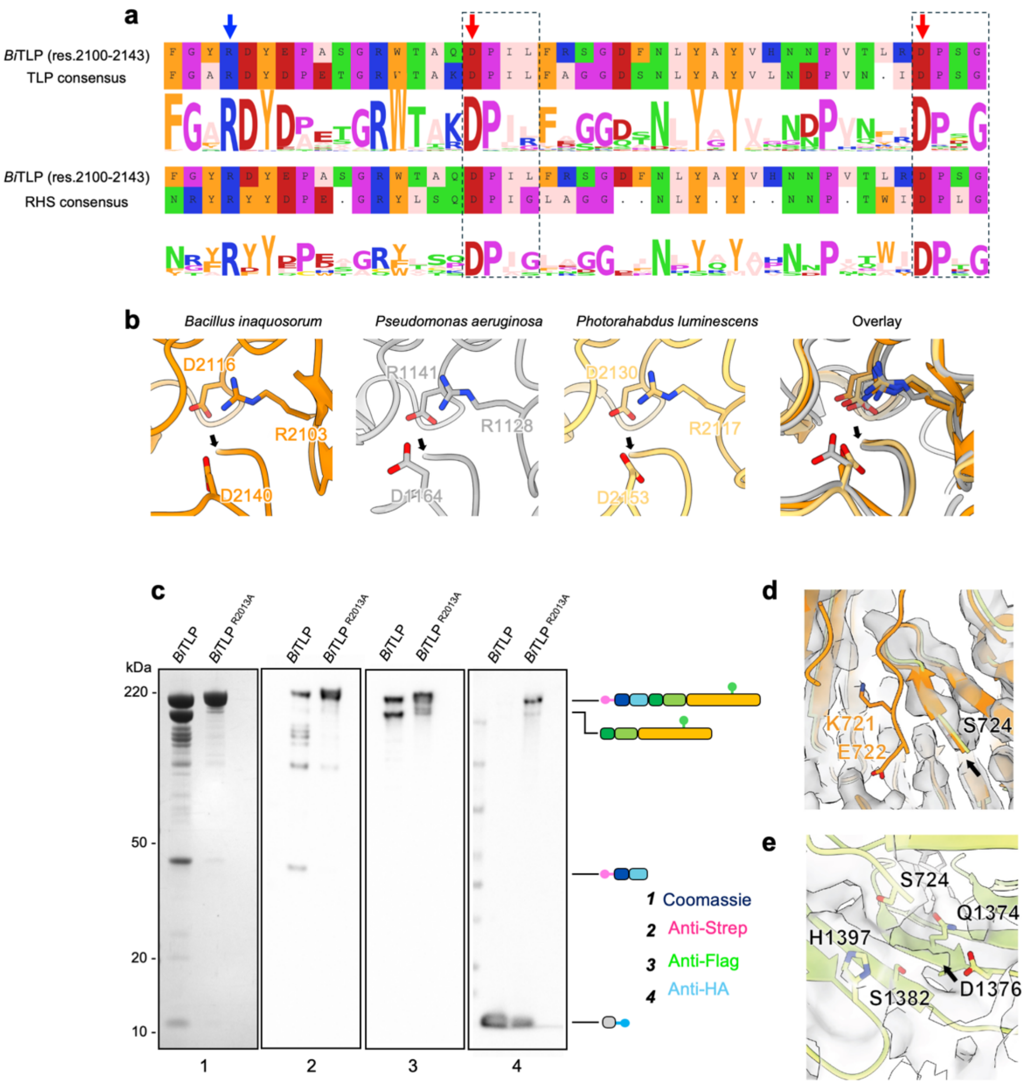
a
BiTLP (res.2100-2143)
TLP consensus
BiTLP (res.2100-2143)
RHS consensus
b
Bacillus inaquosorum
Pseudomonas aeruginosa
Photorahabdus luminescens
Overlay
D2116
R2103
D2140
R1141
R1128
D1164
D2130
R2117
D2153
c
BiTLP
BiTLP R2013A
kDa
220
50
20
10
1
2
3
4
1 Coomassie
2 Anti-Strep
3 Anti-Flag
4 Anti-HA
d
K721
E722
S724
e
S724
Q1374
H1397
S1382
D1376

Supplementary Fig. 5 | The *Bi*TLP autoproteolytic sites

a Comparison of *Bi*TLP RHS associated core with a consensus sequence derived either from comparison of all TLP sequences described here (top) or from a selection of other RHS proteins : *RhsA, Pseudomonas protegens* [4]. Tse5 , *Pseudomonas Aeruginosa* [5], Tse15, *Acinetobacter Baumannii* [6], Rhs1, *Photorhabdus laumondii* [7], Rhs, *Salmonella bongori* [8], RhsP *Vibrio parahaemolyticus* [9], TcdB2-TccC3, *Photorhabdus luminescens* [10], YenC, *Yersinia entomophaga* [11] (bottom). The sequence logo was generated using the MSAclass logo web server [3]. The bipartite DPxG motifs are boxed in dashed lines. The conserved catalytic aspartate and arginine are highlighted by red and blue arrows. **b** Comparison of *Bacillus inaquosorum* TLP, *Pseudomonas aeruginosa* Tse5 and *Photorhabdus luminescences* TcC autocatalytic sites. Note the presence of a conserved arginine residue (R2103 in *B.inaquosorum*). The aspartyl cleavage sites are indicated with a black arrowhead. **c** SDS-PAGE and western blot analysis of purified *Bi*TLP and the R2103A mutant (*Bi*TLP^{R2103A}): Coomassie staining (1), anti-Strep (2), anti-Flag (3) and anti-HA (4). **d** Overlay of the two *Bi*TLP models: truncated (orange) and full-length (green**).** The absence of visible density upstream of the cleavage site is indicated by an arrow. **e** Focused view of the N-terminal cleavage site, revealing additional density (black arrow) that may correspond to a metal ion, coordinated by surrounding residues.

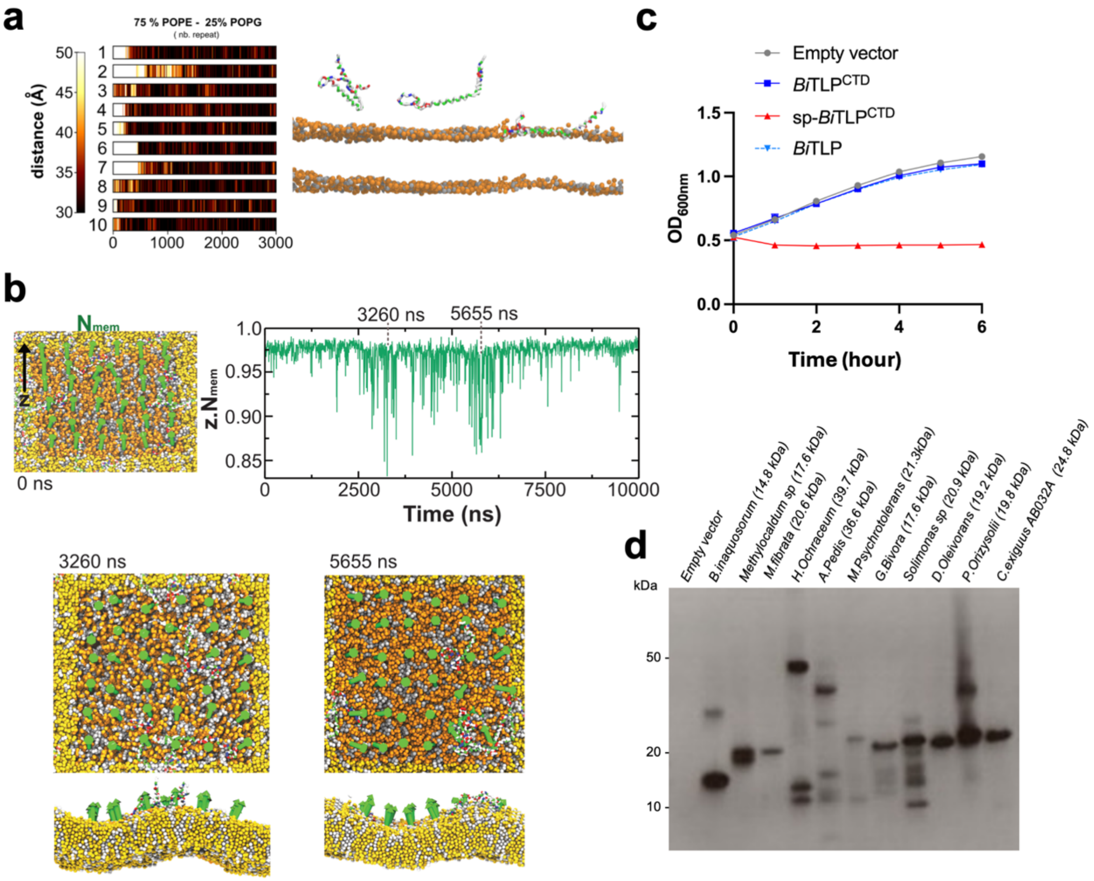
a
75 % POPE - 25% POPG
(nb. repeat)
distance (Å)
50
45
40
35
30
1
2
3
4
5
6
7
8
9
10
0
1000
2000
3000
b
N_{mem}
Z
0 ns
3260 ns
5655 ns
1.0
0.95
0.90
0.85
$z.N_{mem}$
0
2500
5000
7500
10000
Time (ns)
3260 ns
5655 ns
c
Empty vector
BiTLP^{CTD}
sp-BiTLP^{CTD}
BiTLP
OD_{600nm}
1.5
1.0
0.5
0.0
0
2
4
6
Time (hour)
d
kDa
50
20
10
Empty vector
B.inaquosorum (14.8 kDa)
Methylocaldum sp (17.6 kDa)
M.fibrata (20.6 kDa)
H.Ochraceum (39.7 kDa)
A.Pedis (36.6 kDa)
M.Psychrotolerans (21.3kDa)
G.Bivora (17.6 kDa)
Solimonas sp (20.9 kDa)
D.Oleivorans (19.2 kDa)
P.Orizysolii (19.8 kDa)
C.exiguus AB032A (24.8 kDa)

Supplementary Fig. 6 | *Bacillus inaquosorum* CTD binds to the inner membrane and induces membrane deformation.

a Coarse-grained simulation of Bacillus inaquosorum CTD membrane recognition. Simulation was performed with 10 independent calculations of 3 μs. **b** Top: Quantification of membrane curvature based on the dot product between the Z axis and the normal vectors associated to different area of the membrane during the 10 μs CG-MD simulation. A dot product close to one represents a planar membrane. Bottom: Representation of membrane normal vectors and peptide transient association at the membrane at 3260 ns and 5655 ns times. This transient association corresponds to strong membrane deformations. **c** Growth curve of *E. coli* cells expressing *Bacillus inaquosorum* TLP (*Bi*TLP), CTD (*Bi*TLP^{CTD}) or the CTD fused to a N-terminal pelB signal peptide (sp-*Bi*TLP^{CTD}). pBAD empty vector was used as a control. Optical density at 600 nm was measured every hour for 6 hours following induction with 2% L-Arabinose. Points show mean ± SEM, n=3 replicates. **d** Anti-HA immunoblot of affinity-purified samples following protein expression in *E. coli* cells. Each putative 'toxic' CTD was fused with an N-terminal twin-strep-HA tag. The calculated molecular weight of each CTD construct is displayed in brackets.

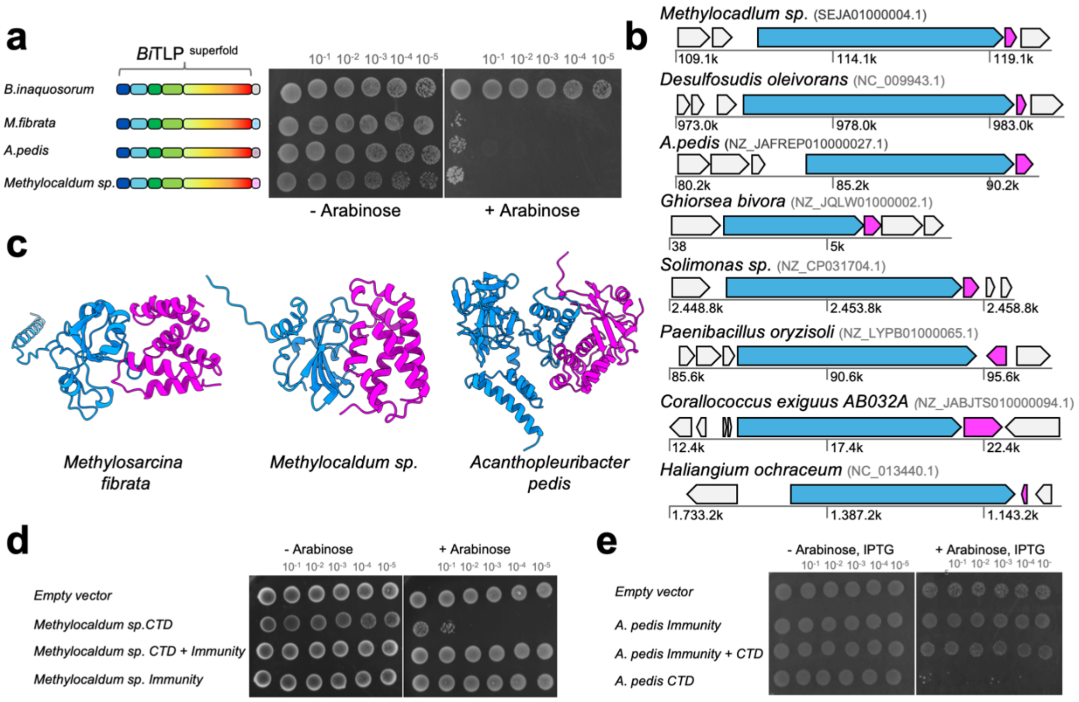
a
BiTLP superfold
B.inaquosorum
M.fibrata
A.pedis
Methylocaldum sp.
- Arabinose
+ Arabinose
b
Methylocadlum sp. (SEJA01000004.1)
Desulfosudis oleivorans (NC_009943.1)
A.pedis (NZ_JAFREP010000027.1)
Ghiorsea bivora (NZ_JQLW01000002.1)
Solimonas sp. (NZ_CP031704.1)
Paenibacillus oryzisoli (NZ_LYPB01000065.1)
Corallococcus exiguus AB032A (NZ_JABJTS010000094.1)
Haliangium ochraceum (NC_013440.1)
c
Methylosarcina fibrata
Methylocaldum sp.
Acanthopleuribacter pedis
d
Empty vector
Methylocaldum sp.CTD
Methylocaldum sp. CTD + Immunity
Methylocaldum sp. Immunity
e
- Arabinose, IPTG
+ Arabinose, IPTG
Empty vector
A. pedis Immunity
A. pedis Immunity + CTD
A. pedis CTD

Supplementary Fig. 7 | Bacterial TLP family members are encoded as effector/immunity pairs.

a Toxicity assays in *E. coli* Top10 cells expressing chimeric protein consisting of *B.inaquosorum* superfold fused with either *M. fibrata*, *Methylocaldum sp*. or *A.pedis* CTD. Bacterial cell cultures were serially diluted on soft agar and incubated overnight at 37°C. To induce gene expression, 2% L-arabinose was added to the medium. **b** Genome analysis of bacterial TLP representatives with their genomic neighborhood. The TLP gene (blue) precedes a smaller ORF which could be its putative immunity gene (magenta). Surrounding genes are colored in grey. **c** Examples of bacterial TLP CTD (blue) heterodimerisation with their corresponding immunity (magenta) proteins, predicted by AlphaFold (Jumper et al. 2021), are shown. **d** The *Methylocadum sp.* putative immunity gene restore *E. coli* growth when co-expressed with the toxin domain. As panel **a** but tested with different *Methylocaldum sp.* constructs: the CTD, the immunity gene or both. **e** As for panel **a** and **d**, but experiments were conducted in *E. coli* BL21 AI expressing *A.pedis* constructs. Protein expression was induced by the addition of arabinose and IPTG.

Supplemntary Figure. 3b

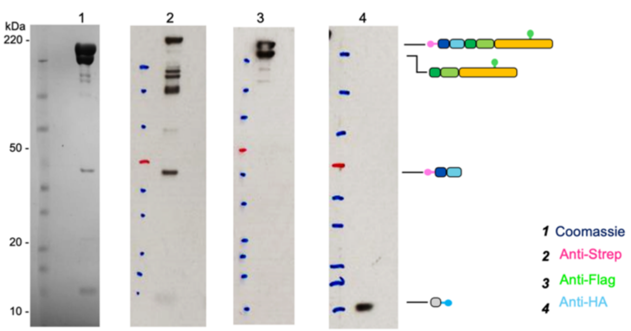

Supplemntary Figure. 5c

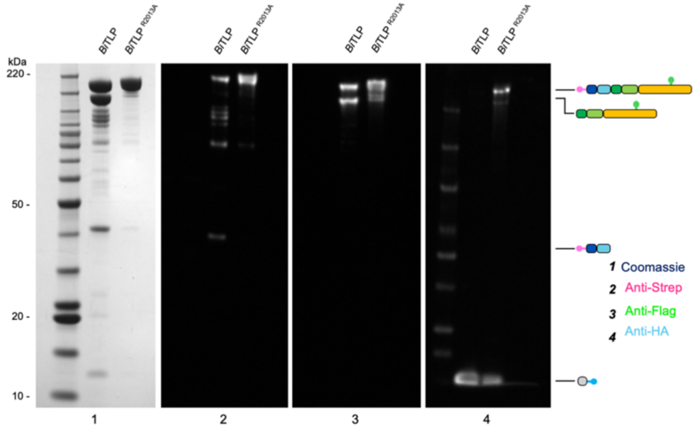

Supplemntary Figure. 6d

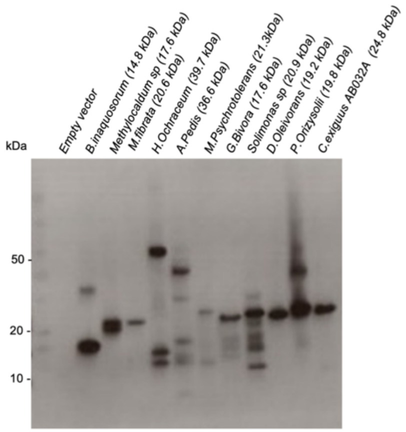

Figure. 5c

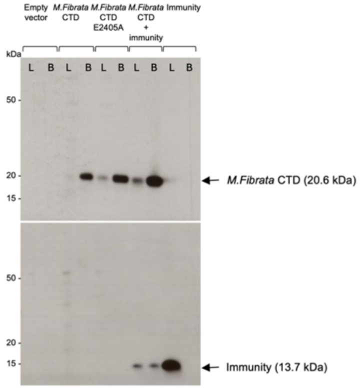

Supplementary Fig. 8 | Unedited Coomassie gels and western blots

Supplementary Table 1 | *Bacillus inaquosorum* TLP (res. 398-2237) and full-length constructs data collection and model refinement statistics table

	Bacillus inaquosorum TLP (res. 398-2237)	*Bacillus inaquosorum* TLP (full length)
**Data collection**		
Microscope	Titan Krios	Cryo-ARM300
Magnification	105000	100000
Voltage (kV)	300	300
Defocus range (um)	-1.5-2.75	-0.5-2.5
Camera	Falcon K3	Gatan K3
Pixel size (A/pixel)	0.832	0.2432
Total electron dose (e-/A2)	38.3	40
Number of images	11017	8721
**Refinement**		
Number of final particles	3,010,268	80,378
Final resolution (A) FSC 0.143 (masked/unmasked)	1.95/2.06	3.83/3.74
Symmetry	C1	C1
Map sharpening B factor (A^2)	-50	-121.8
**Model composition**		
Non-hydrogen atoms	11106	9684
Protein residues	1462	1290
RMSD bonds	0.004	0.007
RMSD angles	0.519	1.011
Model-to-map fit, CC Mask	0.89	0.74
**Validation**		
MolProbity	1.70	1.24
Clash score	5.62	1.00
EMRinger score	5.69	3.48
Poor rotamers (%)	2.42	0.00
**Ramachandran**		
Favoured (%)	97.52	93.00
Allowed (%)	2.48	7.00
Outliers (%)	0.00	0.00

Supplementary Table 2 | Plasmids and primers used in this study

Plasmid	Description	Sources
petMCN-*Bi*TLP	*Bi*TLP res.398-2237 cloned into a pet MCN vector carrying a N-terminal His tag and a tev cleavage site	This study
pProExHta-*BiT*LPFL	*Bi*tLP res.1-2237 cloned into a pProExHta vector carrying a N-terminal His-tag	This study
petMCN-*Bi*TLP (R2103A)	*Bi*TLP res.398-2237 cloned into a pet MCN vector carrying a N-terminal His tag and a tev cleavage with arginine 2103 mutated to Alanine	This study
pBAD-*Bi*TLPCTD	C-terminal domain of *Bi*TLP cloned into a pBAD24 vectors carrying a N-terminal twinstrep-HA tag	This study
pBAD-sp-*Bi*TLPCTD	C-terminal domain of *Bi*TLP cloned into a pBAD24 vectors carrying a pelB signal peptide and a N-terminal twinstrep-HA tag	This study
pBAD-M*ethylocaldum* CTD	C-terminal domain of *Methylocaldum* TLP cloned into a pBAD vector carrying a N-terminal twinstrep-HA tag	This study
pBAD-*M.fibrata* CTD	C-terminal domain of *M.fibrata* TLP cloned into a pBAD vector carrying a N-terminal twinstrep-HA tag	This study
pBAD-*H.ochraceum* CTD	C-terminal domain of *H.ochraceum* TLP cloned into a pBAD vector carrying a N-terminal twinstrep-HA tag	This study
pBAD-*A.pedis* CTD	C-terminal domain of *A.pedis* TLP cloned into a pBAD vector carrying a N-terminal twinstrep-HA tag	This study
pBAD-*M.psychrotolerans* CTD	C-terminal domain of *M.psychrotolerans* TLP cloned into a pBAD vector carrying a N-terminal twinstrep-HA tag	This study
pBAD-*G.bivora* CTD	C-terminal domain of *G.bivora* TLP cloned into a pBAD vector carrying a N-terminal twinstrep-HA tag	This study
pBAD-*Solimonas* CTD	C-terminal domain of *Solimonas* TLP cloned into a pBAD vector carrying a N-terminal twinstrep-HA tag	This study
pBAD-*D.oleivorans* CTD	C-terminal domain of *D.oleivorans* TLP cloned into a pBAD vector carrying a N-terminal twinstrep-HA tag	This study
pBAD-*P.orizysolii* CTD	C-terminal domain of *P.orizysolii* TLP cloned into a pBAD vector carrying a N-terminal twinstrep-HA tag	This study
pBAD-*C.exiguus* AB032A CTD	C-terminal domain of *C.exiguus* AB032A TLP cloned into a pBAD vector carrying a N-terminal twinstrep-HA tag	This study
pBAD-*M.fibrata* CTD (E2405A)	C-terminal domain of *M.fibrata* TLP cloned into a pBAD vector carrying a N-terminal twinstrep-HA tag with Glutamate 2405 mutated to Alanine	This study
pBAD-*M.fibrata* CTD x Immunity	C-terminal domain of *M.fibrata* TLP and the immunity gene are cloned into a pBAD vector carrying a N-terminal twinstrep-HA tag, the Immunity carries a N-terminal Flag tag	This study
pBAD-*M.fibrata* Immunity	*M.fibrata* immunity gene cloned into a pBAD vector carrying a N-terminal Flag tag	This study
pBAD-*Methylocaldum* CTD x Immunity	C-terminal domain of *Methylocaldum* TLP and the immunity gene are cloned into a pBAD vector carrying a N-terminal twinstrep-HA tag, the Immunity carries a N-terminal Flag tag	This study
pBAD-*Methylocaldum* Immunity	*Methylocaldum* immunity gene cloned into a pBAD vector carrying a N-terminal Flag tag	This study
pCDF-*A.pedis* CTD	C-terminal domain of *A.pedis* TLP cloned into a pBAD vector carrying a N-terminal twinstrep-HA tag	This study
petMCN-*A.pedis* immunity	*A.pedis* immunity gene cloned into a petMCN vector carrying a N-terminal His tag	This study

**Primers**	**sequence (5' to 3')**	**Description**
*Bi*TLP_F	CTTTACTTCCAGGGCCATATGGGCTGGAGCCACCC GCAGTTCGAAAAAGGCAGCGGTCGGAGAGCCGCC GTGCTGAGAGGACAG	To add N-terminal StrepII tag upstream residue 398
*Bi*TLP_R	GCTAGCTCTAGACTATTAGGATCCttaAGCGTAATC TGGAACATCGTATGGGTAGTCCAGCTCGATGATG GTTGGCTCAGG	To add C-terminal HA tag downstream residue 2237
*Bi*TLP_F1	gactacaaggacgacgacgacaagGGCGGACCTACCCAGGAG ATGGAACTGACC	To add and internal Flag-tag between residue 1743 and 1744
*Bi*TLP_R1	cttgtcgtcgtcgtccttgtagtcGCCGATAGGGGTCCGCAGCA GCCGGTTCAC	To add and internal Flag-tag between residue 1743 and 1744
*Bi*TLP (R2103A) F	GACACCGAACTGGTGCGGTTTGGCTATgcgGACTA CGAGCCCGCCAGCGGCAGATGG	Arginine 2103 mutation to Alanine
*Bi*TLP (R2103A) R	GCCGCTGGCGGGCTCGTAGTCCGCATAGCCAAAC CGCACCAGTTCGGTGTCAGGGTC	Arginine 2103 mutation to Alanine
*Bi*TLP CTD_F	ccatacgatgttccagattacgctggatcccatatgATCTGCCTGAGCA AGGCCGACATCACC	to clone pBAD-*Bi*TLP CTD and pBAD-sp *Bi*TLP CTD
*Bi*TLP CTD_R	aaaacagccaagcttctcgagtcaGTCCAGCTCGATGATGGTTG GCTCAGG	to clone pBAD-*Bi*TLP CTD and pBAD-sp *Bi*TLP CTD
Methylocaldum CTD_F	ccatacgatgttccagattacgctggatcccatatgGCCGGCGAGAAG CTGCCCATCAGCCCCGGCAGC	to clone pBAD-*Methylocaldum* CTD and pBAD-*Methylocaldum* CTD x Immunity
Methylocaldum CTD_R	aaaacagccaagcttctcgagtcaGGGGAAGGGGAACTTGAAC TCGTTGATGGG	to clone pBAD-*Methylocaldum* CTD
M.fibrata CTD_F	ccatacgatgttccagattacgctggatcccatatgTGCCCCTGGTGCT ACGCCGTGGCCGCCATG	to clone pBAD-*M.fibrata* CTD and pBAD-*M.fibrata* CTD x Immunity
M.fibrata CTD_R	aaaacagccaagcttctcgagtcaCCTGATCCTGGTGATGGCCT CGGGGGGGATCAG	to clone pBAD-*M.fibrata* CTD
H.ochraceum CTD_F	ccatacgatgttccagattacgctggatcccatatgGAGGTGCCCCAGT GGCTGCTGGACGGCTTC	to clone pBAD-*H.ochraceum* CTD
H.ochraceum CTD_R	aaaacagccaagcttctcgagtcaCTTGGGCACGGGCTTGCCC AGGCTCTTCCACTC	to clone pBAD-*H.ochraceum* CTD
A.pedis CTD_F	ccatacgatgttccagattacgctggatcccatatgCTGCCCTTCCTGG TGATCCTGGCCATCAAG	to clone pBAD-*A.pedis* CTD
A.pedis CTD_R	aaaacagccaagcttctcgagtcaCTCGAAGCCCCTGCTGCCGT ACTTCTTGAT	to clone pBAD-*A.pedis* CTD
M.psychrotolerans CTD_F	ccatacgatgttccagattacgctggatcccatatgCTGCCCCAGGAGC TGGTGGACTTCAGC	to clone pBAD-*M.psychrotolerans* CTD
M.psychroltolerans CTD_R	aaaacagccaagcttctcgagtcaCTGGCACTCGCAGCCGCTGT TGGCCATGCC	to clone pBAD-*M.psychrotolerans* CTD
G.bivora CTD_F	ccatacgatgttccagattacgctggatcccatatgatcaaggtgaagcccggcgc caacgtg	to clone pBAD-*G.bivora* CTD
G.bivora CTD_R	aaaacagccaagcttctcgagtcagttgcaggtgctgcccttggggtcgtactc	to clone pBAD-*G.bivora* CTD
Solimonas CTD_F	ccatacgatgttccagattacgctggatcccatatgGAGATCGCCATCG TGGTGAACGGCCCCACC	to clone pBAD-*Solimonas* CTD
Solimonas CTD_R	aaaacagccaagcttctcgagtcaCTTGGGGTTGAAGCTGCTCA GGTTGGCGGG	to clone pBAD-*Solimonas* CTD
D.oleivorans CTD_F	ccatacgatgttccagattacgctggatcccatatgGCCGGCTTCGCCA TCGACGCCGGCGGC	to clone pBAD-*D.oleivorans* CTD
D.oleivorans CTD R	aaaacagccaagcttctcgagtcaCTCCTGCAGGGCGTAGCTCC AGCTCTT	to clone pBAD-*D.oleivorans* CTD

P.orizysolii CTD_F	ccatacgatgttccagattacgctggatcccatatgTTCTGCGTGGGCG CCAGCGTGTACGCCGTG	to clone pBAD-*P.orizysolii* CTD
P.orizysolii CTD_R	aaaacagccaagcttctcgagtcaCCACCTCAGGTTGCTGCACA GCTTGGC	to clone pBAD-*P.orizysolii* CTD
C.exiguus AB032A CTD_F	ccatacgatgttccagattacgctggatcccatatgTTCCCCAGCCCCG CCAAGGCCCCCGTGACC	to clone pBAD-*C.exiguus* AB032A CTD
C.exiguus AB032A CTD_R	aaaacagccaagcttctcgagtcaGGGCAGCTTCATCAGGAAC CACTCCTTCAC	to clone pBAD-*C.exiguus* AB032A CTD
M.fibrata CTD (E2405A)_F	GTGATCTTCAACAGCCTGATCCCCCCCGAGGCCAT CACCAGGATCAGGctc	Glutamate E2405 mutation to Alanine
M.fibrata CTD (E2405A)_R	GATCAGGCTGTTGAAGATCACCgCCTGGCCGCCGC CGGGCATGTTGAAGTC	Glutamate E2405 mutation to Alanine
M.fibrata CTD x Immunity_F	tgatgtcgacctgcagaagaggagaaattaaccATGGACTACAAAG ACGATGACGACAAGACC	To join *M.fibrata* CTD and Immunity
M.fibrata CTD x Immunity_R	ctcttctgcaggtcgacatcaCCTGATCCTGGTGATGGCCTCG GGGGGGATCAG	To join *M.fibrata* CTD and Immunity
M.fibrata Immunity_F	tagcaggaggaattcaccatggactacaaagacgatgacgacaagaccggtatg ACCAACGCGCTGCTGCAGCTGCTGGAT	To clone pBAD *M.fibrata* Immunity
M.fibrata Immunity_R	gccaaaacagccaagcttTTACTCGAGCTACAGGCTTTCAA TCAGGCTCAG	To clone pBAD *M.fibrata* Immunity
Methylocaldum CTD x Immunity_F	tgatgtcgacctgcagaagaggagaaattaaccATGGACTACAAAG ACGATGACGACAAGACC	To join *Methylocaldum* CTD and Immunity
Methylocaldum CTD x Immunity_R	ggttaatttctcctcttctgcaggtcgacatcaGGGGAAGGGGAACTT GAACTCGTTGATGGG	To join *Methylocadlum* CTD and Immunity
Methylocaldum Immunity_F	tagcaggaggaattcaccatggactacaaagacgatgacgacaagaccggtatg GATCGCGAAAAATGGATTAGCGGCATTCTG	To clone pBAD *Methylocaldum* Immunity
Methylocaldum Immunity_R	gccaaaacagccaagcttCTAAATGCACTGGCTCAGTTCAT GCAGCAC	To clone pBAD *Methylocaldum* Immunity
A.pedis CTD_F1	ccatacgatgttccagattacgctcatatgCTGCCCTTCCTGGTGAT CCTGGCCATCAAG	To clone pCDF-*A.pedis* CTD
A.pedis CTD_R1	agctctagactattaggatcctcaTCACTCGAAGCCCCTGCTGC CGTACTTCTT	To clone pCDF-*A.pedis* CTD
A.pedis immunity_F	CTTTACTTCCAGGGCCATATGgactacaaagacgatgacgaca agaccggtatgacccaaaagaaccaagcagacctgcaa	To clone petMCN-*A.pedis* Immunity
A.pedis immunity_R	GCTAGCTCTAGACTATTAGGATCCtcacggcttcacctcggt caggtgcag	To clone petMCN-*A.pedis* Immunity

Supplementary References

1. Jolley, K. A., Bray, J. E. & Maiden, M. C. J. Open-access bacterial population genomics: BIGSdb software, the PubMLST.org website and their applications. *Wellcome Open Research* **3**, 124 (2018).
2. Jumper, J. *et al.* Highly accurate protein structure prediction with AlphaFold. *Nature* **596**, 583–589 (2021).
3. González-Reyes, M. F., Durán-Verdugo, F., Valdés-Jiménez, A. & Riedelsberger, J. MSA Class Logos: a web server for automated sequence logo generation for user-defined sequence classes based on one multiple sequence alignment. *Bioinformatics Advances* **5**, vbaf217 (2025).
4. Günther, P. *et al.* Structure of a bacterial Rhs effector exported by the type VI secretion system. *PLOS Pathogens* **18**, e1010182 (2022).
5. González-Magaña, A. *et al.* Structural and functional insights into the delivery of a bacterial Rhs pore-forming toxin to the membrane. *Nat Commun* **14**, 7808 (2023).
6. Hayes, B. K. *et al.* Structure of a Rhs effector clade domain provides mechanistic insights into type VI secretion system toxin delivery. *Nat Commun* **15**, 8709 (2024).
7. Jurėnas, D. *et al.* Mounting, structure and autocleavage of a type VI secretion-associated Rhs polymorphic toxin. *Nat Commun* **12**, 6998 (2021).
8. Kielkopf, C. S., Shneider, M. M., Leiman, P. G. & Taylor, N. M. I. T6SS-associated Rhs toxin-encapsulating shells: Structural and bioinformatical insights into bacterial weaponry and self-protection. *Structure* **0**, (2024).

9. Tang, L. *et al.* Vibrio parahaemolyticus prey targeting requires autoproteolysis-triggered dimerization of the type VI secretion system effector RhsP. *Cell Reports* **41**, 111732 (2022).

10. Gatsogiannis, C. *et al.* Tc toxin activation requires unfolding and refolding of a β-propeller. *Nature* **563**, 209–213 (2018).

11. Busby, J. N., Panjikar, S., Landsberg, M. J., Hurst, M. R. H. & Lott, J. S. The BC component of ABC toxins is an RHS-repeat-containing protein encapsulation device. *Nature* **501**, 547–550 (2013).
