# Supplementary material for: Ancestral neuronal receptors are bacterial accessory toxins
Source: Nat Commun. 2026 Feb 14;17:2753. doi: 10.1038/s41467-026-69246-x (PMC13018210; doi:10.1038/s41467-026-69246-x)
Supplement: Supplementary file 2 — Description of Additional Supplementary Files [file 41467_2026_69246_MOESM2_ESM.pdf]

## Description of Additional Supplementary Files

**Supplementary data 1** | List of bacterial species containing TLP genes, identified by analysing the contents of the PubMLST Multispecies database (separate file)

**Supplementary data 2** | List of bacterial species resulting from analysis of the PubMLST Multispecies database using profile Hidden Markov Models (HMM) constructed for the domains of the Teneurin superfold (FN-plug, NHL and YD-shell) (separate file)

**Supplementary data 3** | Bacterial TLP sequences (in fasta format) resulting from analysis of the PubMLST Multispecies database using profile Hidden Markov Models (HMM) constructed for the domains of the Teneurin superfold (FN-plug, NHL and YD-shell) (separate file)

**Supplementary data 4** | Bacillus inaquosorum TLP protein sequence with cleavage sites annotated (based on structural and Nterminal Edman sequencing results) (separate file) The full-length sequence of BiTLP is colored based on domain composition: Nterminal domain (black), TTR (dark blue), TIP (aqua blue), FN-plug (forest green), NHL (lime green), YD-shell (orange), and CTD (light grey). Residues identified from the Nterminal sequencing experiments carried out by AltaBioscience are highlighted in yellow. The two catalytic cleavage sites are indicated by black arrows.

**Supplementary data 5** | TLPs contain a hypervariable Cterminal region (separate file). Multiple sequence alignment of TLP C-termini, calculated using MUSCLE and visualized with Jalview 1,2. The alignment uses the default ClustalX coloring scheme 3.

**Supplementary data 6** | Selected sequences of TLP associated “immunity” genes (in fasta format) (separate file)
