# Supplementary material for: Ancestral neuronal receptors are bacterial accessory toxins
Source: Nat Commun. 2026 Feb 14;17:2753. doi: 10.1038/s41467-026-69246-x (PMC13018210; doi:10.1038/s41467-026-69246-x)
Supplement: Supplementary file 6 — Supplementary Data 4 [file 41467_2026_69246_MOESM6_ESM.pdf]

*Bi*TLP

```
MEERPSPPHL VALTIIPASI VLDVGETHQF QVMGNFSDGS SKDLTKRARY KSSNSNIVKV  60
NNSSHNKGLA TAITAGESEI TARVRQFKVV ANIRVQAAVN LTGITIEPTS VILPVGNTQQ  120
FTVTGQFSDG STQDLTDQAS YLSSSTNVVT VYNNGLATAV SSGTAQITAA VNGFTAIASL  180
EVQAAVTLTG ITIEPTSVIL PAGNTQQFTV TGQFSDGSTQ DLTDQASYLS SNTNVVTVDN  240
NGLATAVSSG TAQITAAVNS FTAIASLEVQ AAVTLIGITI EPTSVVLKVE ETQQFTVTGR  300
FSDGSTQDVT EQASYASSNP NVVTIANTGL ATAVALGSAT ITATADGFTA IATLNVHTIV  360
VPPLDMSGAT SVFSSSAFLY TGENPIQTGV QPGTIELRRA AVLRGQVFNR DGEPLSRVNI  420
SILDHPEFGS TFTREDGQFD MAVNGGELLI VRYEKNGLRP VQRRIEVPWE DFVILPDVQM  480
IALDPVVTTI DLSQPDIQTA RGSEILDVEG TRQATLLFPP GNKATMILPD GTTQEISTLN  540
VRATEYSVGE GGPNAMPALL PPTSAYTYCV EFSADEELAA GAREVRFDQP IVFYVENFLE  600
FP[illegible]GAVPTG FYDRIQGEWI ASRNGQVIQI VSITGGLAD LDIDGDGAAD SADDLAELGIT  660
NA[illegible]QRLANL YQTGQSLWRV PITHFSAPWD CNWPYGFPDD SDRPRNPDPF DKPKPDDDCN  720
KEGSIIGALA QTLGEEVQVT GTPFRMHYHS DRVRGRKEAY SLEIPLSGTN IPQSVQRIRL  780
DIFVAGRCIT ESFPPATNLT HTFVWDGKDA YGRVLQGSHP ITVRIRYEYQ LVYLTPAAFR  840
TSFGRITGEG GGSGGGGAGT PLIIARRGDP NASLTQEFKG SIGLFDTREQ GLGAWTLSVH  900
HFYDPISRVL FLGDGQRRSA ESLSTVIATV AGTNYGFSGD GGPATQAQLR APRDMAVGSD  960
GSLYIADTEN ERIRRVGPDG IITTVAGTGV QGFSGDGGPA TQAQLGSPRG VAVGSDGSLY  1020
IVDAGNVRIR RVGPDGIITT VAGTGVSGFS GDGGPATQAQ LSFPPGGVAV GSDGSLFIAD  1080
TLNNRIRRVG PDGIITTVAG TGDFGFSGDG GPAAQATLRI PGDVSVGSDG SLYIADSQNV  1140
RIRRVGPDGI INTVAGTGVQ GFSGDGGPAT QAQLRLPRGV DVGSDGYLYI VDESRTRRVR  1200
DGIITTVVGT GVQGFSGDGG PATQATLWVP ADVAVGSDGS LFIADTGNNR IRRVASVLPG  1260
TTRTDILIPS ADGSEVVIFN ESGKHLRTLD ALTGAIRFRF IYNNDGHLVQ VQDVDGNSTI  1320
IERDSTGNPI SIVAPGGQRT ALTLDANGFL ASITNPAREA FQFEYNPDGL MTSQIDPRGN  1380
ISRFEYDSGG HLIRDEHPTG GVTTLMRTNS TNGFVVTLTS PLGRVSTFQL ERLTTGTLKQ  1440
VVIDSNGGRT ESLTGTDGKQ QITYPDGTQL VDQVGPDPRF GMLAHIVRRR TVTTPGGLSF  1500
LHVTDRQAVL SDPTNLLSLQ KLTTTVSIND RIFRTIFDAG TRETTITTPV GRKSVIGFDS  1560
IGRVNRQILA TGVDPILFTY NNQGQLTERQ QGNVITNLIY DSLLRLQAIV DNAGRESRFS  1620
YDNADRVIQI TRCGGDIERL TYDSNGNPTQ VIRPNGSVHT LSYTPVNLLG GYTPPGNPGY  1680
TFLYNVERQI RRKILPTGRT IDLTYDSGGR LTDVIYPEAA VTLVYTAGDP TQRVNRLLRT  1740
PIGGGPTQEM ELTYDASLIT GMTFTGISQG AFTYTYDSNF SLVNVGLVSG SDQVQVGISR  1800
NADGLITGLG SFTITRSGPD GKISRISDGA LNRTMSYDTI ARLSSYNDTV GGQQIYRSDF  1860
QYDNASRLQR KTETVGSAAH TLEYSYDTSC NLIEVTKDGM VVESYTYDAN GNRTSRQVMG  1920
GPVEMATYDN QDRLVHRDGI NYEFNADGFM VSRGSDTFEY SALGELLQAT VGGKTITYVY  1980
DGLGRRVSRT ESTGTTQYLY GNPENLLQVT AIRDPSGQLN MLF[illegible]DNDFL FAFDRDGTKF  2040
YVTTDLVGTP RVVTNGTGTV LRELEHDSFG NIIADSNPRF VL[illegible]FAGGL ADPDTELVRF  2100
GYRDYEPASG RWTAQDPILF RSGDFNLYAY VHNNPVTLRD PSGLICLSKA DITTLKEIND  2160
VIKIVGAVAT AGGFLFANPY ATAAGIGISL GGAINSLIID AIDECPQEPP KPANKCPERQ  2220
PVNFKRDSPE PTIIELD                                                2237
```

Alta Bioscience Ltd
37 Walkers Road
Manor Side
Redditch
B98 9HE
United Kingdom

Tel: +44 (0) 1527 584495
info@altabioscience.com
www.altabioscience.com

UKAS TESTING 2370

Protein sequencing cover sheet

Sheet printed 09 February 2023

Analysis for:-

Finaritra Raoelijaona<br>University of Oxford<br>University of Oxford<br>Department of Biochemistry<br>South Parks Road<br>Oxford<br>OX1 3QU	Analysis codes S7653<br>S7654<br><br>Date logged in 09 February 2023<br>by:- Marion Peak

Analysed following Standard Operating Procedure # 271 "Scope of protein sequencing analysis"

Procedures used Protein on PVDF blot. Following SOP#190, 'washblot'

This report set contains 3 pages *Including this one*

Analyst / technician ☐<br>Technical manager ☐<br>Quality manager ☐<br>Director ☑	Data approved and released by [signature]<br>Issue Date ~~0~~ 15 /02/ 23
Quality manager ☐<br>Deputy quality manager ☐	QA approval ____________<br>Date ___/___/___<br>*required for GMP samples only*

These samples have been analysed in compliance with ISO 17025:2017 and ISO 9001:2015

AltaBioscience welcomes customer feedback as a means of improving its operation and services. In order to monitor our service to you, we would be grateful if you provide any feedback using our webform at https://altabioscience.com/customer-feedback/
Details of the quality system are shown on:- www.altabioscience.com

Opinions: All opinions expressed in the enclosed reports are outside the scope of any accreditations.
Copies: All reproductions of the report data must be in full.

Note: Whilst AltaBioscience has exercised all due care in the producing of these documents, it is on the strict understanding that AltaBioscience, its servant and/or agents shall not be legally liable for any error, mistake or inaccuracy which any opinion or advice or conclusion may contain, either under Common Law or as a result of any breach of duty arising under contract or otherwise.

2730

Protein sequence report

Created on 15-Feb-23

Alta Bioscience code: S7653

Customer sample code: SMALL N-term ~ 10kDa

N terminus

Residue			
1	**I**	P ?	
2	**-**		
3	**L**		
4	**S**		
5	**K ?**	S ?	
6			
7			
8			
9			
10			
11			
12			
13			
14			
15			

Comments:- Evidence of possible 2nd sequence.

Notes on the presentation of the data:-
? = most probable assignment. **-** = nothing detected at this position. X = unknown component
Where several sequences are observed, an attempt is made to arrange them in descending order of abundance at each residue. However because of difficulties inherent in the sequencing process, this should be treated as a guide only. These results only relate to the sample tested. Tests are performed at AltaBioscience premises.

Reported by [signature] Date 15/02/23

File name: S7653 F Raoelijaona.docx
Template: Proseq15, version 7. last modified 18th January 2023

For conditions of sale, please refer to our website at www.altabioscience.com

Protein sequence report

Created on 15-Feb-23

Alta Bioscience code: S7654

Customer sample code: LARGE N-term ~ 160kDa

N terminus

Residue			
1	**S**		
2	**I**		
3	**-**		
4	**G**		
5	**A**		
6			
7			
8			
9			
10			
11			
12			
13			
14			
15			

Comments:- Very low signal

Notes on the presentation of the data:-

? = most probable assignment. - = nothing detected at this position. X = unknown component

Where several sequences are observed, an attempt is made to arrange them in descending order of abundance at each residue. However because of difficulties inherent in the sequencing process, this should be treated as a guide only. These results only relate to the sample tested. Tests are performed at AltaBioscience premises.

Reported by [signature] Date 15/02/23

File name: S7654 F Raoelijaona.docx

Template: Proseq15, version 7. last modified 18th January 2023

For conditions of sale, please refer to our website at www.altabioscience.com

Alta Bioscience Ltd
37 Walkers Road
Manor Side
Redditch
B98 9HE
United Kingdom

Tel: +44 (0) 1527 584495
Fax: +44 (0) 1527 67368
info@altabioscience.com
www.altabioscience.com

Delivery note

Note:
To ensure prompt payment, please forward this delivery note to the appropriate accounts office.

Work done for

Finaritra Raoelijaona

University of Oxford
Department of Biochemistry
South Parks Road
Oxford
OX1 3QU

Your reference	AL2127503
Your account code	UOXNBIO
Print date	09 February 2023

Type of work	Protein Sequencing
Client name	Finaritra Raoelijaona
Alta code	S7653
Alta code	S7654
Alta code	
Alta code	
Alta code	

In case of query:
Contact Accounts section
T: +44 (0) 1527 584495
E: accounts@altabioscience.com

For terms and conditions see :- www.altabioscience.com
