# Supplementary material for: Ancestral neuronal receptors are bacterial accessory toxins
Source: Nat Commun. 2026 Feb 14;17:2753. doi: 10.1038/s41467-026-69246-x (PMC13018210; doi:10.1038/s41467-026-69246-x)
Supplement: Supplementary file 7 — Supplementary Data 5 [file 41467_2026_69246_MOESM7_ESM.pdf]

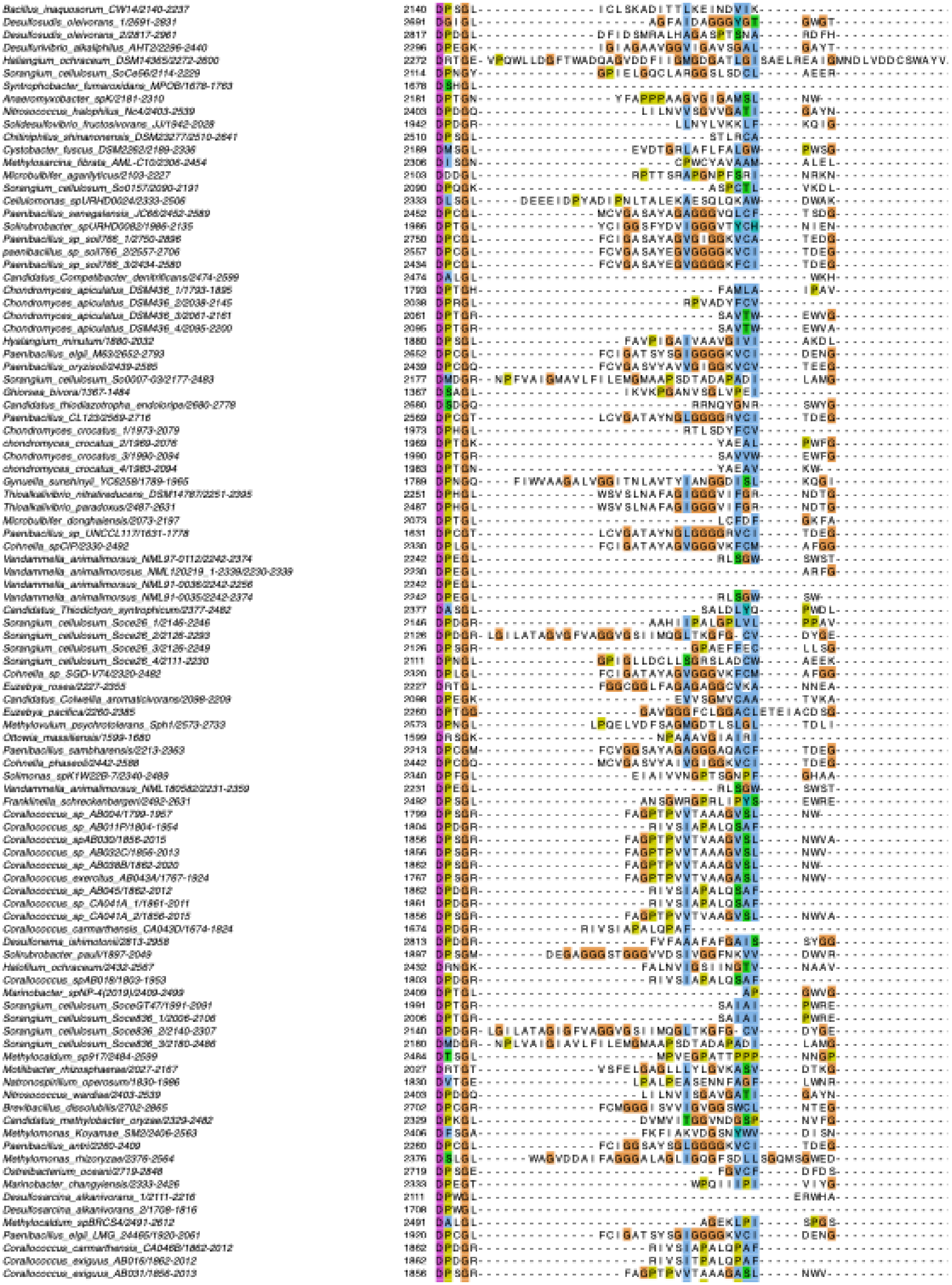

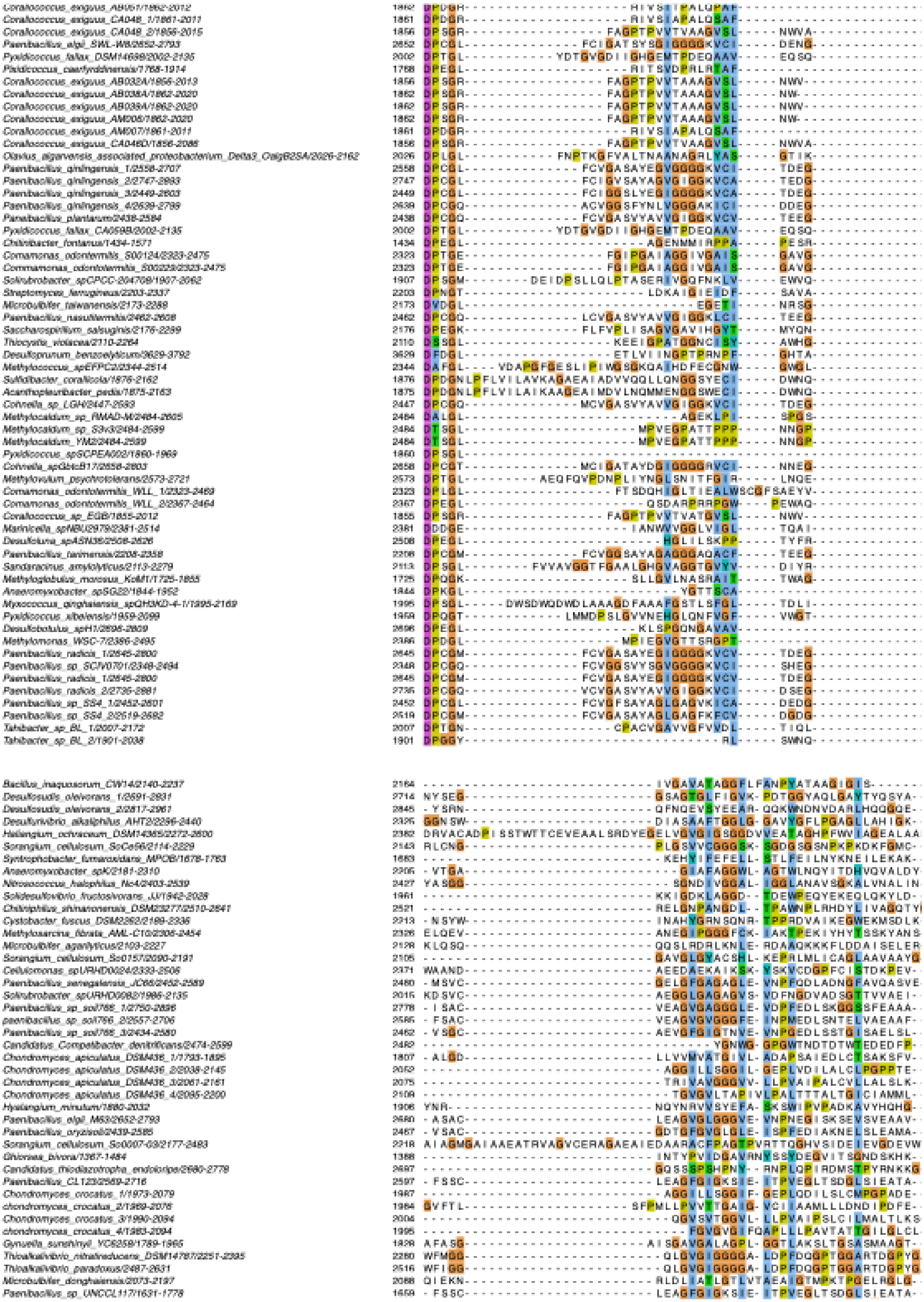

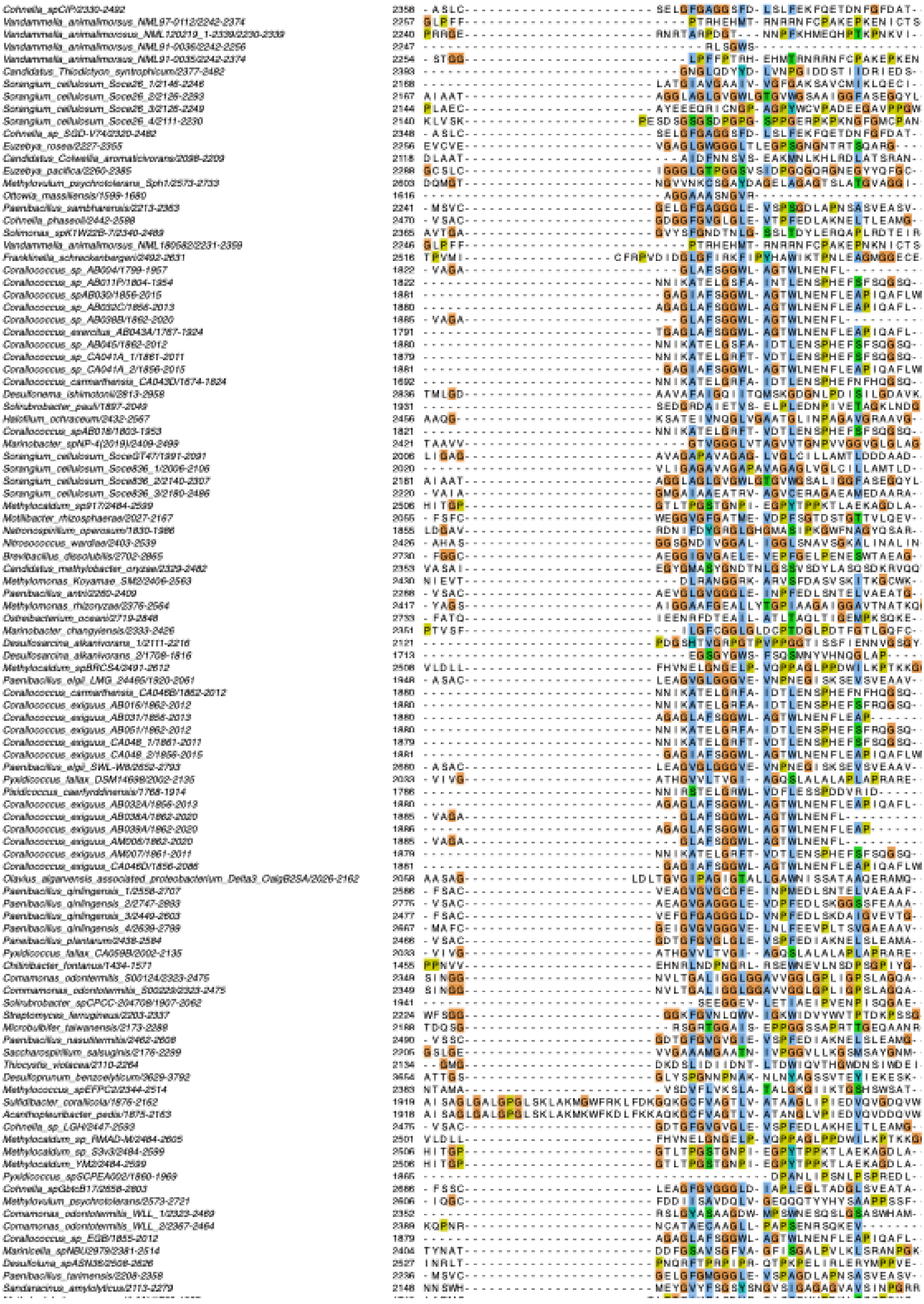

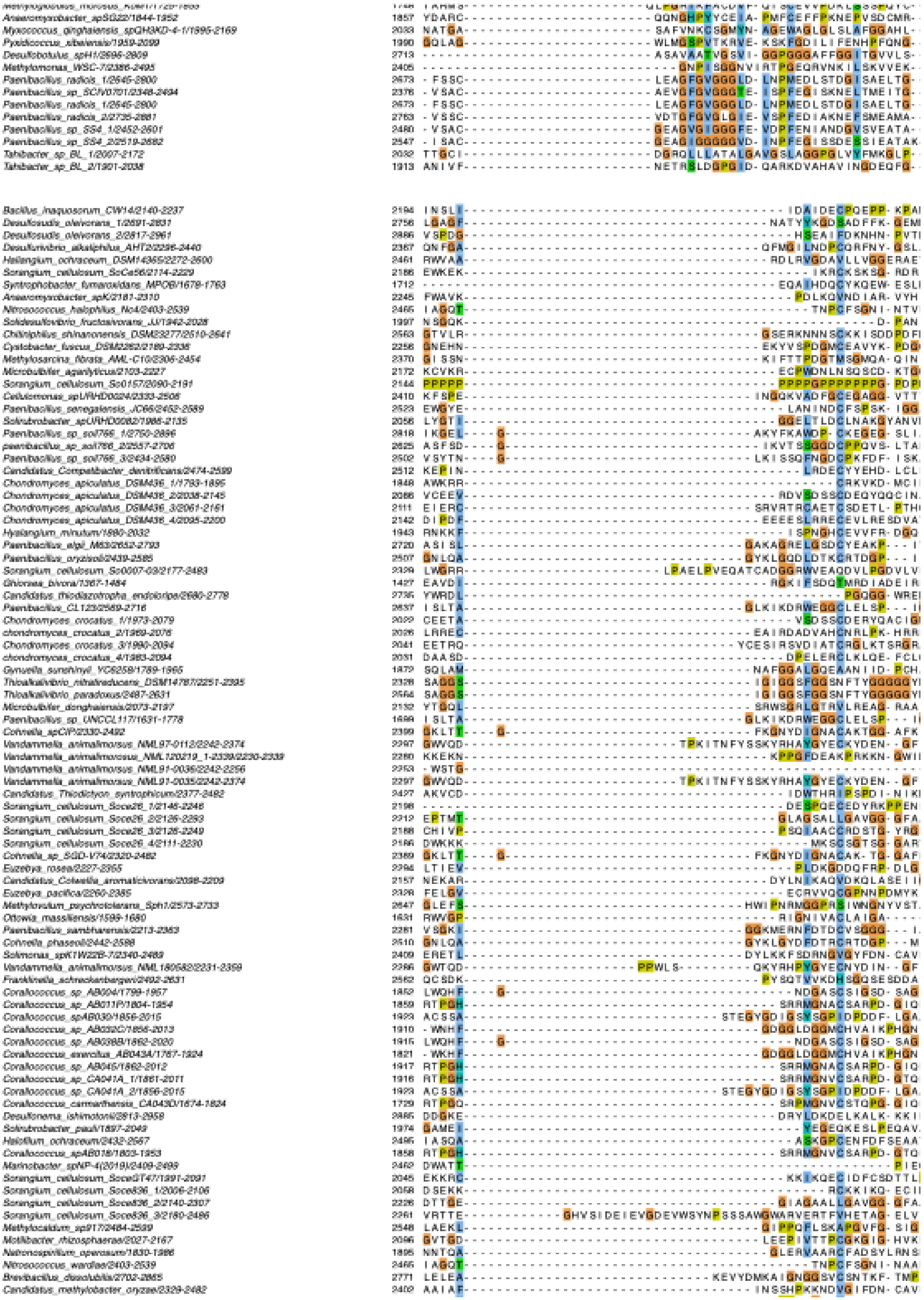

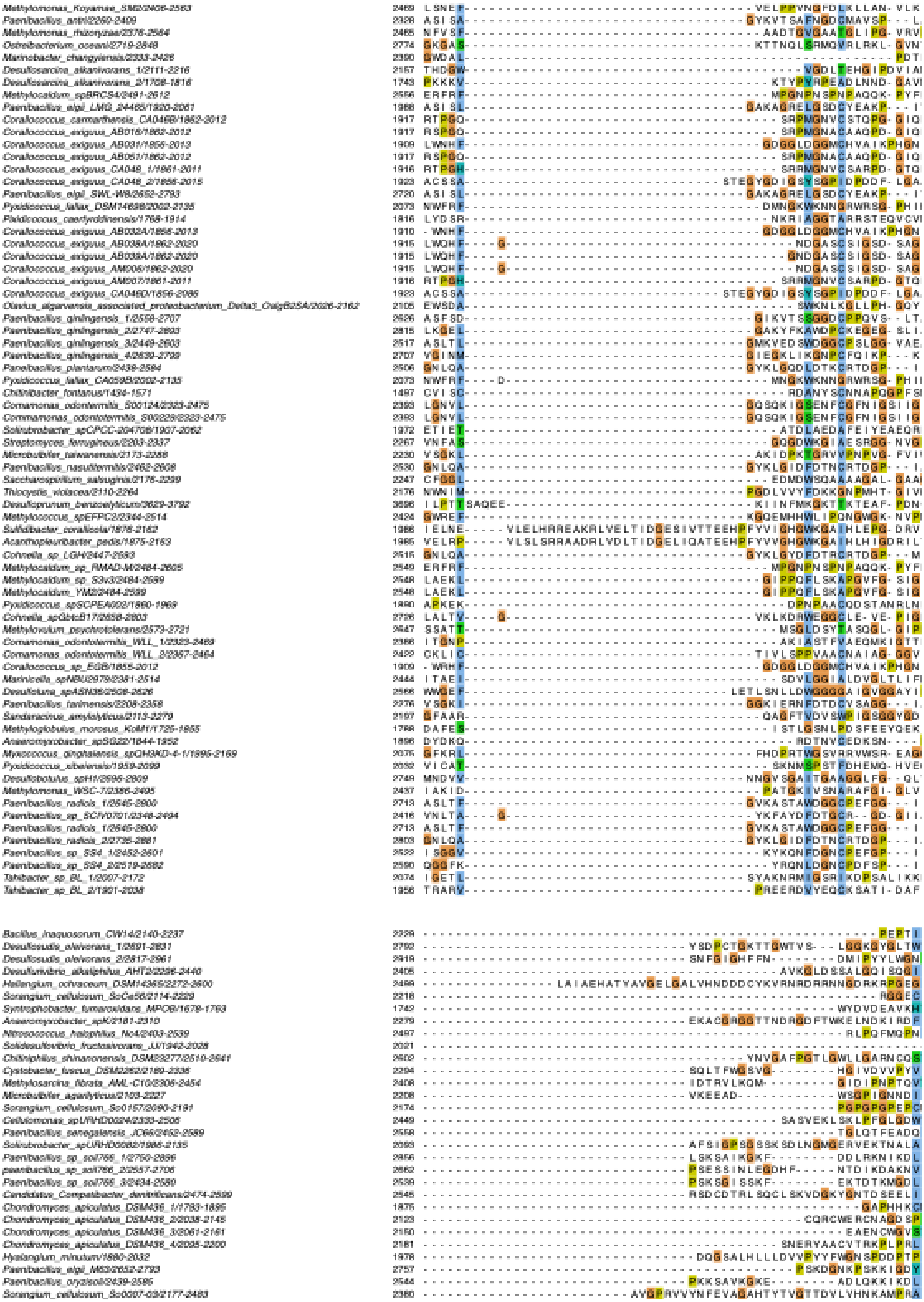

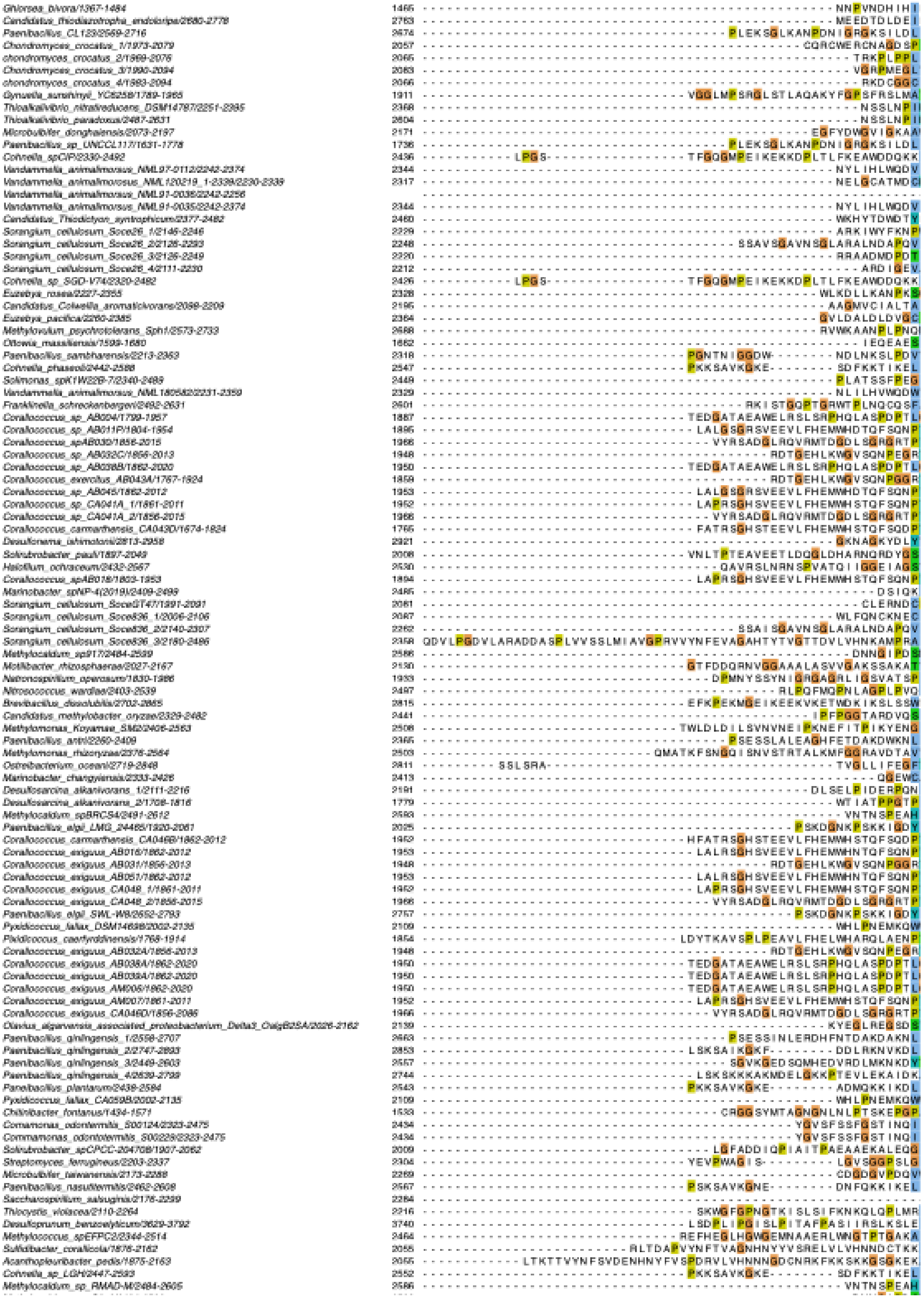

Methylocaldum_sp_53v3/2484-2599	2586	---------- DNNGIPDS
Methylocaldum_YM2/2484-2599	2586	---------- DNNGIPDS
Pyxidicoccus_spSCPEA002/1860-1969	1926	---------- GLRRAWDFATTQDKT
Cohnella_spGbtcB17/2658-2803	2763	---------- PLEKFSVKGKE-------- ENLGKNILDL
Methylovulum_psychrotolerans/2573-2727	2681	---------- VTPVVDGGV---------- VAPSNRYNGG
Comamonas_odontotermitis_WLL_1/2323-2469	2423	---------- GGHEGYQQYHGRFELDHFLMDI
Comamonas_odontotermitis_WLL_2/2367-2464		----------
Corallococcus_sp_EGB/1855-2012	1947	---------- RDTGEHLKWGVSQNPEGR
Marinicella_spNBU2979/2381-2514	2481	---------- EHTIEGDHEHGLMIYICSGSGDC
Desulfoluna_spASN36/2509-2626	2609	---------- DIDTRGVYDPVNNP
Paenibacillus_tarimensis/2208-2358	2313	---------- PGNSNAGGDW-------- KDLNKSLPDI
Sandaracinus_amylolyticus/2113-2279	2236	---------- GGEIDFHGAIGKTWAFPHGHIDF
Methyloglobulus_morosus_KoM1/1725-1855	1826	---------- PGFLDPSPKERSQDQNEFGDA
Anaeromyxobacter_spSG22/1844-1952	1926	---------- WEHESCLASC
Myxococcus_qinghaiensis_spQH3KD-4-1/1995-2169	2114	---------- QRWGNVNAGFNYMPLTASFNSWMNGSSA
Pyxidicoccus_xibeiensis/1959-2099	2069	---------- LQTYSRLTTG
Desulfobotulus_spH1/2696-2809	2788	---------- YGIDLMAYGGDP
Methylomonas_WSC-7/2386-2495	2475	---------- LNGDSLADNA
Paenibacillus_radicis_1/2645-2800	2750	---------- LKKSGAKGKE-------- SQLKHDVRDL
Paenibacillus_sp_SCIV0701/2348-2494	2453	---------- PEESAVSGEN-------- DSMKSKVKDL
Paenibacillus_radicis_1/2645-2800	2750	---------- LKKSGAKGKE-------- SQLKHDVRDL
Paenibacillus_radicis_2/2735-2881	2840	---------- PKKSAVKGNE-------- DNFQKTIKEL
Paenibacillus_sp_SS4_1/2452-2601	2557	---------- PSKSGRKFKLDGLKGGPSWDLKNIKPGL
Paenibacillus_sp_SS4_2/2519-2682	2624	---------- RGLSENVGPFNLNVTDPGKSGLKFKIDG
Tahibacter_sp_BL_1/2007-2172	2115	---------- GTYFVESKFGSAKLTGPQRAAAKK
Tahibacter_sp_BL_2/1901-2038	1995	---------- FNMRRNDSRKPFQGHEIETQNGPFENS

Bacillus_inaquosorum_CW14/2140-2237		----------
Desulfosudis_oleivorans_1/2691-2831	2827	YALQE----------
Desulfosudis_oleivorans_2/2817-2961	2956	YSGPC---------P----------
Desulfurivibrio_alkaliphilus_AHT2/2296-2440	2436	SLGNL----------
Haliangium_ochraceum_DSM14365/2272-2600	2583	TAKQAYEEWKSLGKPVPK----------
Sorangium_cellulosum_SoCe56/2114-2229	2225	QCTAQ----------
Syntrophobacter_fumaroxidans_MPOB/1678-1763	1761	PCM----------
Anaeromyxobacter_spK/2181-2310		----------
Nitrosococcus_halophilus_Nc4/2403-2539	2535	AGRLF----------
Solidesulfovibrio_fructosivorans_JJ/1942-2028	2024	SCPDK----------
Chitiniphilus_shinanonensis_DSM23277/2510-2641	2637	RQDLD----------
Cystobacter_fuscus_DSM2262/2189-2336	2333	PAAP----------
Methylosarcina_fibrata_AML-C10/2306-2454	2444	LIPPE---------AITRIR----------
Microbulbifer_agarilyticus/2103-2227		----------
Sorangium_cellulosum_So0157/2090-2191	2188	ACCE----------
Cellulomonas_spURHD0024/2333-2506	2499	ADQNYSNP----------
Paenibacillus_senegalensis_JC66/2452-2589	2583	VCGQW---------NW----------
Solirubrobacter_spURHD0082/1986-2135	2131	HCWQY----------
Paenibacillus_sp_soil766_1/2750-2896	2890	QCLQY---------KW----------
paenibacillus_sp_soil766_2/2557-2706	2700	ACAQY---------KW----------
Paenibacillus_sp_soil766_3/2434-2580	2574	GCAQY---------KW----------
Candidatus_Competibacter_denitrificans/2474-2599	2592	RPHLN---------YWD----------
Chondromyces_apiculatus_DSM436_1/1793-1895	1893	--GVE----------
Chondromyces_apiculatus_DSM436_2/2038-2145	2142	RCGF----------
Chondromyces_apiculatus_DSM436_3/2061-2161		----------
Chondromyces_apiculatus_DSM436_4/2095-2200		----------
Hyalangium_minutum/1880-2032	2026	IPEELWR----------
Paenibacillus_algi_M63/2652-2793	2787	ACGSM---------RW----------
Paenibacillus_oryzisoli/2439-2595	2579	LCSNL---------RW----------
Sorangium_cellulosum_So0007-03/2177-2483	2454	LNDKYGPGNYDKGAGSEFSKIKKWGDRSFE----------
Ghiorsea_bivora/1367-1484	1482	TCN----------
Candidatus_thiodiazotropha_endoloripes/2680-2778	2777	-CY----------
Paenibacillus_CL123/2569-2716	2710	GCKSY---------KW----------
Chondromyces_crocatus_1/1973-2079	2076	KCLF----------
chondromyces_crocatus_2/1969-2076		----------
Chondromyces_crocatus_3/1990-2094		----------
chondromyces_crocatus_4/1983-2094	2089	LPGDYN----------
Gynuella_sunshinyii_YC6258/1789-1965	1961	FGGPF----------
Thioalkalivibrio_nitratireducens_DSM14787/2251-2395	2391	VIGWW----------
Thioalkalivibrio_paradoxus/2487-2631	2627	VIGWW----------
Microbulbifer_donghaiensis/2073-2197	2193	ECEGN----------
Paenibacillus_sp_UNCCL117/1631-1778	1772	GCKSY---------KW----------
Cohnella_spCIP/2330-2492	2487	TCARLY----------
Vandammella_animalmorsus_NML97-0112/2242-2374	2370	TEVGR----------
Vandammella_animalmorsus_NML120219_1-2339/2230-2339	2332	PCKGWVPK----------
Vandammella_animalmorsus_NML91-0036/2242-2256		----------
Vandammella_animalmorsus_NML91-0035/2242-2374	2370	TEVGR----------
Candidatus_Thiodictyon_syntrophicum/2377-2482	2478	LNYRF----------
Sorangium_cellulosum_Soce26_1/2146-2246	2243	-CGGV----------
Sorangium_cellulosum_Soce26_2/2126-2293	2288	SNGPSE----------
Sorangium_cellulosum_Soce26_3/2126-2249	2245	QAQGF----------
Sorangium_cellulosum_Soce26_4/2111-2230	2226	QCLVQ----------
Cohnella_sp_SGD-V74/2320-2482	2477	TCARL---------Y----------
Euzebya_rosea/2227-2355	2349	RCVTYYY----------
Candidatus_Colwellia_aromaticivorans/2089-2209		----------
Euzebya_pacifica/2260-2385	2381	VCFPL----------
Methylovulum_psychrotolerans_Sph1/2573-2733	2729	GCECQ----------
Ottowia_massiliensis/1599-1680	1676	IPGGP----------
Paenibacillus_sambharensis/2213-2363	2357	VCGQY---------RW----------
Cohnella_phaseoli/2442-2588	2582	YCSNL---------RW----------
Solimonas_spK1W22B-7/2340-2489	2485	SFNPK----------
Vandammella_animalmorsus_NML180582/2231-2359	2355	TEVGH----------
Franklinella_schreckenbergeri/2492-2631	2628	SCRR----------
Corallococcus_sp_AB004/1799-1957	1941	HPGLD---------TFGIIKFASGKP----------
Corallococcus_sp_AB011P/1804-1954	1950	FNANP----------
Corallococcus_spAB030/1856-2015	2011	IPLSP----------
Corallococcus_sp_AB032C/1856-2013	1992	RDMVELDPGPLNHEPWAGKGLP----------
Corallococcus_sp_AB038B/1862-2020	2004	HPGLD---------TFGIIKFASGKP----------
Corallococcus_exercitus_AB043A/1767-1924	1903	RDMVELDPGPLNHEPWAGKGIP----------
Corallococcus_sp_AB045/1862-2012	2008	FNANP----------
Corallococcus_sp_CA041A_1/1861-2011	2007	FNANP----------
Corallococcus_sp_CA041A_2/1856-2015	2011	IPLSP----------
Corallococcus_carmarthensis_CA043D/1674-1824		----------
Desulfonema_ishimotonii/2813-2958	2954	NIYNL----------
Solirubrobacter_pauli/1897-2049	2045	AEIGM----------
Halolium_ochraceum/2432-2567	2563	GGAAF----------

Corallococcus_spAB018/1802-1953	1949	FNANP-
Marinobacter_spNP-4(2019)/2409-2499	2497	SCE-
Sorangium_cellulosum_SoceGT47/1891-2081		-
Sorangium_cellulosum_Soce836_1/2006-2106	2101	DCPRDS-
Sorangium_cellulosum_Soce836_2/2140-2307	2302	SNGPSK-
Sorangium_cellulosum_Soce836_3/2180-2486	2442	RPYQD- - - - - - - - - - - - - - ESGKEFAERLLNEKYGPGNFDKGPGSEFNQIRKWGDRSFE-
Methylocaldum_sp917/2484-2599	2598	PC-
Motilibacter_rhizosphaerae/2027-2167		-
Natronospirillum_operosum/1830-1986	1982	ILGAD-
Nitrosococcus_wardiae/2403-2539	2535	MGRLF-
Brevibacillus_dissolubilis/2702-2865	2859	LCFAV- - - - - - - - - - - - - - KW-
Candidatus_methylobacter_oryzae/2329-2482	2476	KFNPQ-
Methylomonas_Koyamae_SM2/2406-2563	2554	PPSPL- - - - - - - - - - FRKNW-
Paenibacillus_antri/2260-2409	2403	GCAQY- - - - - - - - - - - - - - KW-
Methylomonas_rhizoryzae/2376-2564	2560	PCDCP-
Ostreibacterium_oceani/2719-2848	2844	GCGCE-
Marinobacter_changyiensis/2333-2426	2423	QCGF-
Desulfosarcina_alkanivorans_1/2111-2216	2213	LCGK-
Desulfosarcina_alkanivorans_2/1709-1816	1812	SRCDS-
Methylocaldum_spBRCS4/2491-2612		-
Paenibacillus_algi_LMG_24465/1920-2061	2055	ACGSM- - - - - - - - - - - - - - RW-
Corallococcus_carmarthensis_CA046B/1862-2012	2008	FNANP-
Corallococcus_exiguus_AB016/1862-2012	2008	FNAAP-
Corallococcus_exiguus_AB031/1856-2013	1992	RDMVELDPGPLNHEPWAGKGLP-
Corallococcus_exiguus_AB051/1862-2012	2009	NAAP-
Corallococcus_exiguus_CA048_1/1861-2011	2007	FNANP-
Corallococcus_exiguus_CA048_2/1856-2015	2011	IPLSP-
Paenibacillus_algi_SWL-W8/2652-2793	2787	ACGSM- - - - - - - - - - - - - - RW-
Pyxidicoccus_fallax_DSM14698/2002-2135	2131	KPPKE-
Pixidicoccus_caerfyrddinensis/1768-1914	1910	VFDVP-
Corallococcus_exiguus_AB032A/1856-2013	1992	RDMVELDPGPLNHEPWAGKGLP-
Corallococcus_exiguus_AB038A/1862-2020	2004	HPGLD- - - - - - - - - - - - - - TFGIIKFASGKP-
Corallococcus_exiguus_AB039A/1862-2020	2017	SGKP-
Corallococcus_exiguus_AM006/1862-2020	2004	HPGLD- - - - - - - - - - - - - - TFGIIKFASGKP-
Corallococcus_exiguus_AM007/1861-2011	2007	FNANP-
Corallococcus_exiguus_CA046D/1856-2086	2011	IPMKLRAIIKSKTVKSMGKPVASIEIAIEEGALYLFRQGVNGEPLGDTWHQNLEEAISQA
Olavius_algarvensis_associated_proteobacterium_Delta3_OalgB2SA/2026-2162	2158	GTLMW-
Paenibacillus_qinlingensis_1/2556-2707	2701	ACAQYKW-
Paenibacillus_qinlingensis_2/2747-2893	2887	QCLQY- - - - - - - - - - - - - - KW-
Paenibacillus_qinlingensis_3/2449-2603	2597	LCKQL- - - - - - - - - - - - - - RW-
Paenibacillus_qinlingensis_4/2639-2799	2795	ISSKK-
Panibacillus_plantarum/2438-2584	2578	LCSNL- - - - - - - - - - - - - - RW-
Pyxidicoccus_fallax_CA059B/2002-2135	2131	KPPKE-
Chitinibacter_fontanus/1434-1571	1565	RTLFKMR-
Comamonas_odontermitis_S00124/2323-2475	2471	QAGKK-
Commamonas_odontotermitis_S00229/2323-2475	2471	QAGKK-
Solirubrobacter_spCPCC-204708/1907-2062	2049	TARQRYQEMGIYGK-
Streptomyces_ferrugineus/2203-2337	2334	-FGNK-
Microbulbifer_taiwanensis/2173-2288	2284	MCPAN-
Paenibacillus_nasutitermitis/2462-2608	2602	LCSNL- - - - - - - - - - - - - - RW-
Saccharospirillum_salsuginis/2176-2299	2295	IAEQF-
Thiocystis_violacea/2110-2264	2260	DLFLC-
Desulfoprunum_benzoelyticum/3629-3792	3785	EIFRSFNP-
Methylococcus_spEFPC2/2344-2514	2510	NCECD-
Sulfidibacter_corallicola/1876-2162	2125	DFAKRMMDAQYGEGNWTRKGDMAKQYSQIKKFGDRGFE-
Acanthopleuribacter_pedis/1875-2163	2142	SRKGAQGKQFSQIKKYGSRGFE-
Cohnella_sp_LGH/2447-2593	2587	YCSNL- - - - - - - - - - - - - - RW-
Methylocaldum_sp_RMAD-M/2484-2605		-
Methylocaldum_sp_53v3/2484-2599	2598	PC-
Methylocaldum_YM2/2484-2599	2598	PC-
Pyxidicoccus_spSCPEA002/1860-1969	1965	IPGTF-
Cohnella_spGbtcB17/2658-2803	2797	GCKSY- - - - - - - - - - - - - - KW-
Methylovulum_psychrotolerans/2573-2721	2714	VGAPI- - - - - - - - - - - - - - PVR-
Comamonas_odontotermitis_WLL_1/2323-2469	2466	GCKK-
Comamonas_odontotermitis_WLL_2/2367-2464	2462	ICK-
Corallococcus_sp_EGB/1855-2012	1991	RDMVELDPGPLNHEPWAGKGLP-
Marinicella_spNBU2979/2381-2514	2510	VCIPN-
Desulfoluna_spASN36/2509-2626		-
Paenibacillus_tarimensis/2208-2358	2352	VCGQY- - - - - - - - - - - - - - RW-
Sandaracinus_amylolyticus/2113-2279	2277	PCP-
Methyloglobulus_morosus_KoM1/1725-1855		-
Anaeromyxobacter_spSG22/1844-1952	1948	SQPND-
Myxococcus_qinghaiensis_spQH3KD-4-1/1995-2169	2166	LNDP-
Pyxidicoccus_xibeiensis/1959-2099	2096	RPWP-
Desulfobotulus_spH1/2696-2809	2807	PCY-
Methylomonas_WSC-7/2386-2495		-
Paenibacillus_radicis_1/2645-2800	2794	GCKSF- - - - - - - - - - - - - - RW-
Paenibacillus_sp_SCIV0701/2348-2494	2488	FCRNL- - - - - - - - - - - - - - KW-
Paenibacillus_radicis_1/2645-2800	2794	GCKSF- - - - - - - - - - - - - - RW-
Paenibacillus_radicis_2/2735-2881	2875	VCRNL- - - - - - - - - - - - - - RW-
Paenibacillus_sp_SS4_1/2452-2601	2594	VCGSIRHP-
Paenibacillus_sp_SS4_2/2519-2682	2676	VCAQH- - - - - - - - - - - - - - KW-
Tahibacter_sp_BL_1/2007-2172	2168	AADHP-
Tahibacter_sp_BL_2/1901-2038	2034	LNTYR-
