# Supplementary material for: Ancestral neuronal receptors are bacterial accessory toxins
Source: Nat Commun. 2026 Feb 14;17:2753. doi: 10.1038/s41467-026-69246-x (PMC13018210; doi:10.1038/s41467-026-69246-x)
Supplement: Supplementary file 9 — Reporting Summary [file 41467_2026_69246_MOESM9_ESM.pdf]

nature portfolio

Corresponding author(s): Elena Seiradake

Last updated by author(s): Dec 17, 2025

Reporting Summary

Nature Portfolio wishes to improve the reproducibility of the work that we publish. This form provides structure for consistency and transparency in reporting. For further information on Nature Portfolio policies, see our Editorial Policies and the Editorial Policy Checklist.

Statistics

For all statistical analyses, confirm that the following items are present in the figure legend, table legend, main text, or Methods section.

n/a	Confirmed	
☐	☒	The exact sample size (*n*) for each experimental group/condition, given as a discrete number and unit of measurement
☐	☒	A statement on whether measurements were taken from distinct samples or whether the same sample was measured repeatedly
☐	☒	The statistical test(s) used AND whether they are one- or two-sided<br>*Only common tests should be described solely by name; describe more complex techniques in the Methods section.*
☒	☐	A description of all covariates tested
☐	☒	A description of any assumptions or corrections, such as tests of normality and adjustment for multiple comparisons
☒	☐	A full description of the statistical parameters including central tendency (e.g. means) or other basic estimates (e.g. regression coefficient) AND variation (e.g. standard deviation) or associated estimates of uncertainty (e.g. confidence intervals)
☒	☐	For null hypothesis testing, the test statistic (e.g. *F*, *t*, *r*) with confidence intervals, effect sizes, degrees of freedom and *P* value noted<br>*Give P values as exact values whenever suitable.*
☐	☐	For Bayesian analysis, information on the choice of priors and Markov chain Monte Carlo settings
☒	☐	For hierarchical and complex designs, identification of the appropriate level for tests and full reporting of outcomes
☒	☐	Estimates of effect sizes (e.g. Cohen's *d*, Pearson's *r*), indicating how they were calculated

Our web collection on statistics for biologists contains articles on many of the points above.

Software and code

Policy information about availability of computer code

Data collection	EPU, SerialEM v3.1
Data analysis	Bioinformatic analysis:<br>MAFFT v7.490<br>HMMER v3.1b1<br>EMBOSS transeq v6.6.0.0<br>MUSCLE v3.8.31<br>Jalview v2.11.5.1<br>TrimAL v1.4.1 (NG phylogeny)<br>IqTree (https://iqtree.github.io)<br>Itol v7<br>signal p5.0<br>Interproscan (Interpro)<br><br>Cryo-EM data processing:<br>SIMPLE 3.0<br>CryoSPARC<br>RELION v4-0

Model building and refinement
Coot v.09.8.3
PHENIX 1.20.1-4437
ISOLDE (ChimeraX)
DeepEMhancer
ChimeraX v1.8

Structural prediction and homology search
Alphafold2
DALI server
Foldseek

Propidium iodine staining
FIJI-ImageJ v2.16.0
MICROBE J 3.13p
Graphpad prism v9

Growth curve and biocehmical assay
Graphpad prism v9

For manuscripts utilizing custom algorithms or software that are central to the research but not yet described in published literature, software must be made available to editors and reviewers. We strongly encourage code deposition in a community repository (e.g. GitHub). See the Nature Portfolio guidelines for submitting code & software for further information.

Data

Policy information about availability of data

All manuscripts must include a data availability statement. This statement should provide the following information, where applicable:

- Accession codes, unique identifiers, or web links for publicly available datasets
- A description of any restrictions on data availability
- For clinical datasets or third party data, please ensure that the statement adheres to our policy

The cryo-EM maps have been deposited in the Electron Microscopy Data Bank (EMDB) under accession codes EMD-52847 [https://www.ebi.ac.uk/pdbe/entry/emdb/EMD-52847] (BiTLP); and EMD-71831 [https://www.ebi.ac.uk/pdbe/entry/emdb/EMD-71831] (BiTLPFL). The atomic coordinates have been deposited in the Protein Data Bank (PDB) under accession codes PDB9IFO [https://doi.org/10.2210/pdb9IFO/pdb] (BiTLP); and PDB9PT5 [https://doi.org/10.2210/pdb9PT5/pdb] (BiTLPFL). The data generated in this study are provided in the supplementary files or in the source data file. Unedited gels and Western blots are shown in Supplementary Fig.8.

Research involving human participants, their data, or biological material

Policy information about studies with human participants or human data. See also policy information about sex, gender (identity/presentation), and sexual orientation and race, ethnicity and racism.

Reporting on sex and gender	n/a
Reporting on race, ethnicity, or other socially relevant groupings	n/a
Population characteristics	n/a
Recruitment	n/a
Ethics oversight	n/a

Note that full information on the approval of the study protocol must also be provided in the manuscript.

Field-specific reporting

Please select the one below that is the best fit for your research. If you are not sure, read the appropriate sections before making your selection.

☒ Life sciences ☐ Behavioural & social sciences ☐ Ecological, evolutionary & environmental sciences

For a reference copy of the document with all sections, see nature.com/documents/nr-reporting-summary-flat.pdf

Life sciences study design

All studies must disclose on these points even when the disclosure is negative.

Sample size	No statistical methods were used to predetermine sample size. However, samples used for the growth curve experiments were selected to represent each phyla containing TLPs and were guided by structural homology analyses to inform downstream biochemical assays. Sample selection for biochemical assays and microscopy followed established practices in the literature.
Data exclusions	No data were excluded from the analysis
Replication	Experiments were performed in triplicate from three independent biological samples
Randomization	Experiments were performed using random selection
Blinding	No blinding was applied, as data collection and analysis did not require subjective human intervention

Reporting for specific materials, systems and methods

We require information from authors about some types of materials, experimental systems and methods used in many studies. Here, indicate whether each material, system or method listed is relevant to your study. If you are not sure if a list item applies to your research, read the appropriate section before selecting a response.

Materials & experimental systems

n/a	Involved in the study	
☐	☒	Antibodies
☒	☐	Eukaryotic cell lines
☒	☐	Palaeontology and archaeology
☒	☐	Animals and other organisms
☒	☐	Clinical data
☒	☐	Dual use research of concern
☒	☐	Plants

Methods

n/a	Involved in the study	
☒	☐	ChIP-seq
☒	☐	Flow cytometry
☒	☐	MRI-based neuroimaging

Antibodies

Antibodies used	Primary antibodies: anti-HA (sigma Aldrich, H3663-200UL), anti-strepII (IBA, 2-1507-001) or anti-FLAG (sigma Aldrich, F1804-50UG) , secondary antibody: anti-mouse IgG HRP (ThermoFisher, 31430)
Validation	Anti-HA sigma Aldrich, H3663-200UL) was used for western blotting in Figure 4c and supplementary Fig. 3b, 5c, 6d<br>Anti-strepII (iba, 2-1507-001) was used for western blotting in supplementary Fig. 3c and 5c<br>Anti-FLAG (sigma Aldrich, F1804-50UG)was used for western blotting in supplementary Fig. 3c, 5c and 6d

Plants

Seed stocks	n/a
Novel plant genotypes	n/a
Authentication	n/a
